# An explainable machine learning-based prediction model for sarcopenia in elderly Chinese people with knee osteoarthritis

**DOI:** 10.1007/s40520-025-02931-x

**Published:** 2025-03-07

**Authors:** Ziyan Wang, Yuqin Zhou, Xing Zeng, Yi Zhou, Tao Yang, Kongfa Hu

**Affiliations:** 1 School of Artificial Intelligence and Information Technology, Nanjing University of Chinese Medicine, Nanjing, 210023 China; 2 Institute of Chinese Medicine Literature, Nanjing University of Chinese Medicine, Nanjing, 210023 China; 3 Affiliated Hospital of Nanjing University of Chinese Medicine (Jiangsu Province Hospital of Chinese Medicine), Nanjing, 210029 China; 4 Department of Traumatology and Orthopedics, Wuxi Affiliated Hospital of Nanjing University of Chinese Medicine, Nanjing University of Chinese Medicine, Wuxi, 214071 China; 5 Jiangsu Province Engineering Research Center of TCM Intelligence Health Service, Nanjing, 210023 China

**Keywords:** Sarcopenia, Knee osteoarthritis, CHARLS, Machine learning, Prediction model, SHAP

## Abstract

**Background:**

Sarcopenia is an age-related progressive skeletal muscle disease that leads to loss of muscle mass and function, resulting in adverse health outcomes such as falls, functional decline, and death. Knee osteoarthritis (KOA) is a common chronic degenerative joint disease among elderly individuals who causes joint pain and functional impairment. These two conditions often coexist in elderly individuals and are closely related. Early identification of the risk of sarcopenia in KOA patients is crucial for developing intervention strategies and improving patient health.

**Methods:**

This study utilized data from the China Health and Retirement Longitudinal Study (CHARLS), selecting symptomatic KOA patients aged 65 years and above and analyzing a total of 95 variables. Predictive factors were screened via least absolute shrinkage and selection operator (LASSO) regression and logistic regression. Eight machine learning algorithms were employed to construct predictive models, with internal cross-validation and independent test validation performed. The final selected model was analyzed via the SHapley Additive exPlanations (SHAP) method to enhance interpretability and clinical applicability. To facilitate clinical use, we developed a web application based on this model (http://106.54.231.169/).

**Results:**

The results indicate that six predictive factors—body mass index, upper arm length, marital status, total cholesterol, cystatin C, and shoulder pain—are closely associated with the risk of sarcopenia in KOA patients. CatBoost demonstrated excellent overall performance in both calibration analyses and probability estimates, reflecting accurate and dependable predictions. The final results on the independent test set (accuracy = 0.8902; F1 = 0.8627; AUC = 0.9697; Brier score = 0.0691) indicate that the model possesses strong predictive performance and excellent generalization ability, with predicted probabilities closely aligning with actual occurrence rates and thereby underscoring its reliability.

**Conclusion:**

From the perspective of public health and aging, this study constructed an interpretable sarcopenia risk prediction model on the basis of routine clinical data. This model can be used for early screening and risk assessment of symptomatic KOA patients, assisting health departments and clinicians in the early detection and follow-up of relevant populations, thereby improving the quality of life and health outcomes of elderly individuals.

**Supplementary Information:**

The online version contains supplementary material available at https://doi.org/10.1007/s40520-025-02931-x .

## Introduction

Sarcopenia is a progressive and systemic skeletal muscle disease associated with aging characterized by a decrease in muscle mass and function, resulting in various adverse outcomes, such as falls, functional decline, frailty, and death [1], as well as increased health care costs [2]. With the increasing severity of population aging today, it has become one of the world’s major public health concerns. Studies have indicated that 10–16% of elderly individuals suffer from sarcopenia, and the prevalence of sarcopenia is greater among patients than among the general population [3]. Additionally, there is an increasing incidence of sarcopenia among middle-aged people [1]. Recently, sarcopenia has become more critical in the diagnosis and treatment of this condition. In 2010, the European Working Group on Sarcopenia in Older People (EWGSOP) published the definition of sarcopenia, with the aim of promoting the identification and prognosis of patients with sarcopenia. The most widely used diagnostic criterion for sarcopenia was the EWGSOP, which was updated in 2019 [4]. Sarcopenia is typically diagnosed in Asian populations on the basis of the consensus proposed and updated by the Asian Working Group for Sarcopenia (AWGS) [5].

Knee osteoarthritis (KOA) is a chronic degenerative joint disease characterized by pain, stiffness, and progressive loss of function, affecting quality of life, disability, medication consumption, and mortality [6]. With the aging of the Chinese population, the prevalence of KOA is increasing annually, making it an important clinical issue. A previous nationwide survey revealed that the prevalence of symptomatic KOA among middle-aged and older individuals in China was 8.1%, and it increased with age [7]. Bones and muscles are closely related, and age progressively affects their functions. Several biomechanical factors contribute to the development, progression, and severity of KOA, including changes in skeletal structures and surrounding joint muscles [8].

Although the interaction between KOA and sarcopenia remains unclear, their coexistence and possible relationship cannot be ignored, particularly since muscle exercises have been shown to improve the prognosis and quality of life of KOA patients, particularly elderly individuals. Moreover, studies have demonstrated that sarcopenia is an independent risk factor for KOA [9]. Symptomatic KOA patients usually suffer from muscle weakness, which reflects a strong association between KOA and sarcopenia [10]. Patients with these conditions suffer functional impairment and deterioration of quality of life, increasing the disease burden.

Currently, treatment strategies for symptomatic KOA patients with sarcopenia are still being explored. Studies have shown that short-term aerobic exercise and muscle-strengthening training are promising for reducing knee osteoarthritis pain, improving physical function, and improving quality of life [11]. It is also believed that combining physical therapy for sarcopenia with KOA treatment can provide significant benefits [12]. Thus, identifying sarcopenia in KOA patients is crucial for choosing subsequent treatment and intervention strategies.

**Fig. 1:**
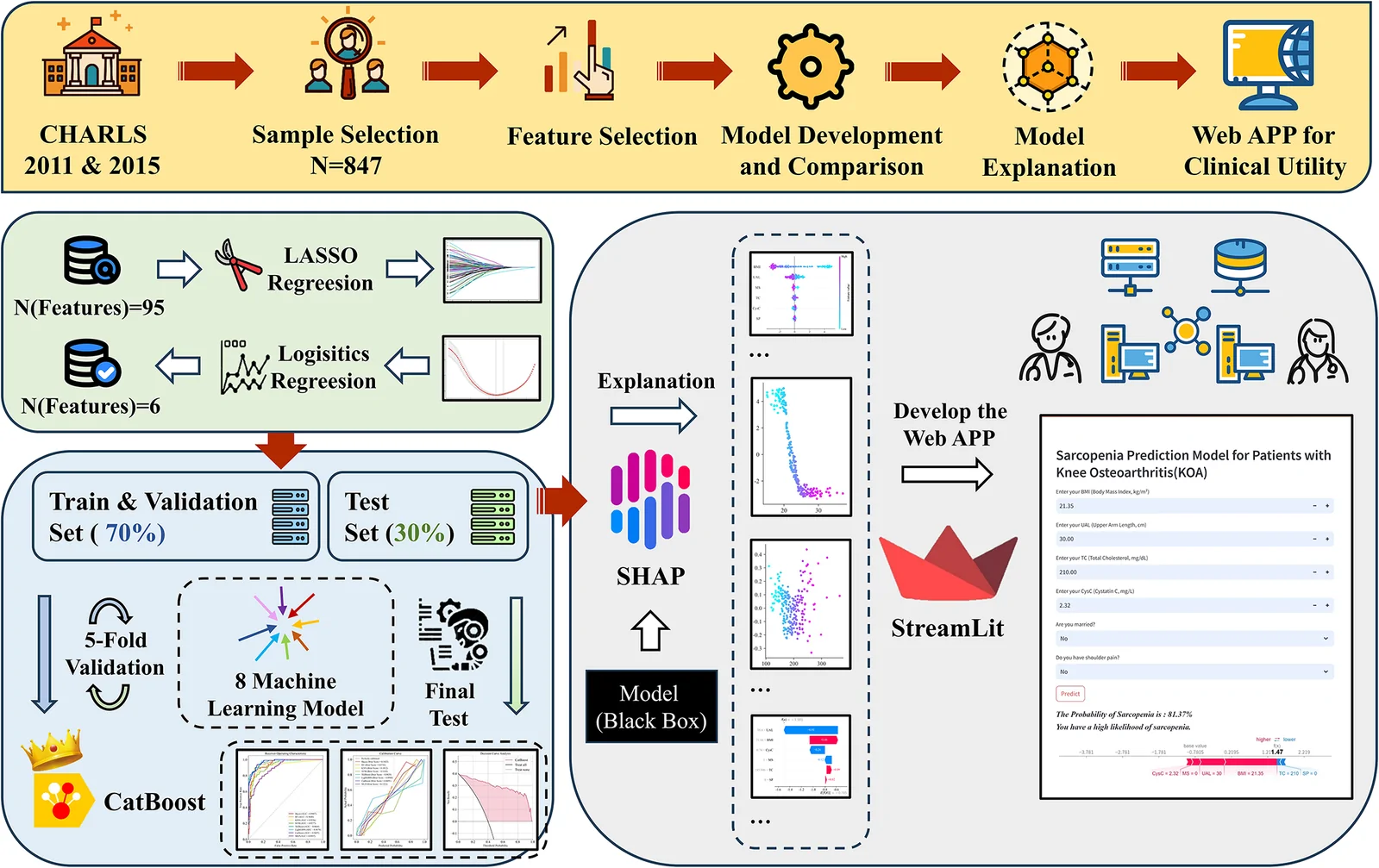
Research framework and visual summary

Several models predict the risk of KOA or sarcopenia among Chinese elderly individuals [13, 14]. However, prediction models for sarcopenia in patients with KOA remain limited. While joint degeneration and muscle loss are prevalent degenerative conditions in elderly individuals, previous models have focused primarily on the risk of sarcopenia in older adults [14, 15] or the risk of KOA alone [16–18]. The coexistence of sarcopenia in KOA patients presents a complex challenge involving multiple social, medical, and clinical factors. Therefore, a single predictor may be insufficient to assess the risk of sarcopenia effectively, particularly compared with other outcome indicators. As a result, in this study, we constructed a comprehensive, multifactorial prediction model for sarcopenia in KOA patients on the basis of national survey data from the China Health and Retirement Longitudinal Study (CHARLS). We aimed to develop a prediction model that can identify the risk of sarcopenia in KOA patients at an early stage utilizing multidimensional data such as demographic, lifestyle habits, and biomarker data, facilitating early management of muscle health in KOA patients and providing a basis for early intervention and personalized treatment. The framework and visual summary of the study are shown in Fig. 1.

## Method

### Population and sample

**Fig. 2:**
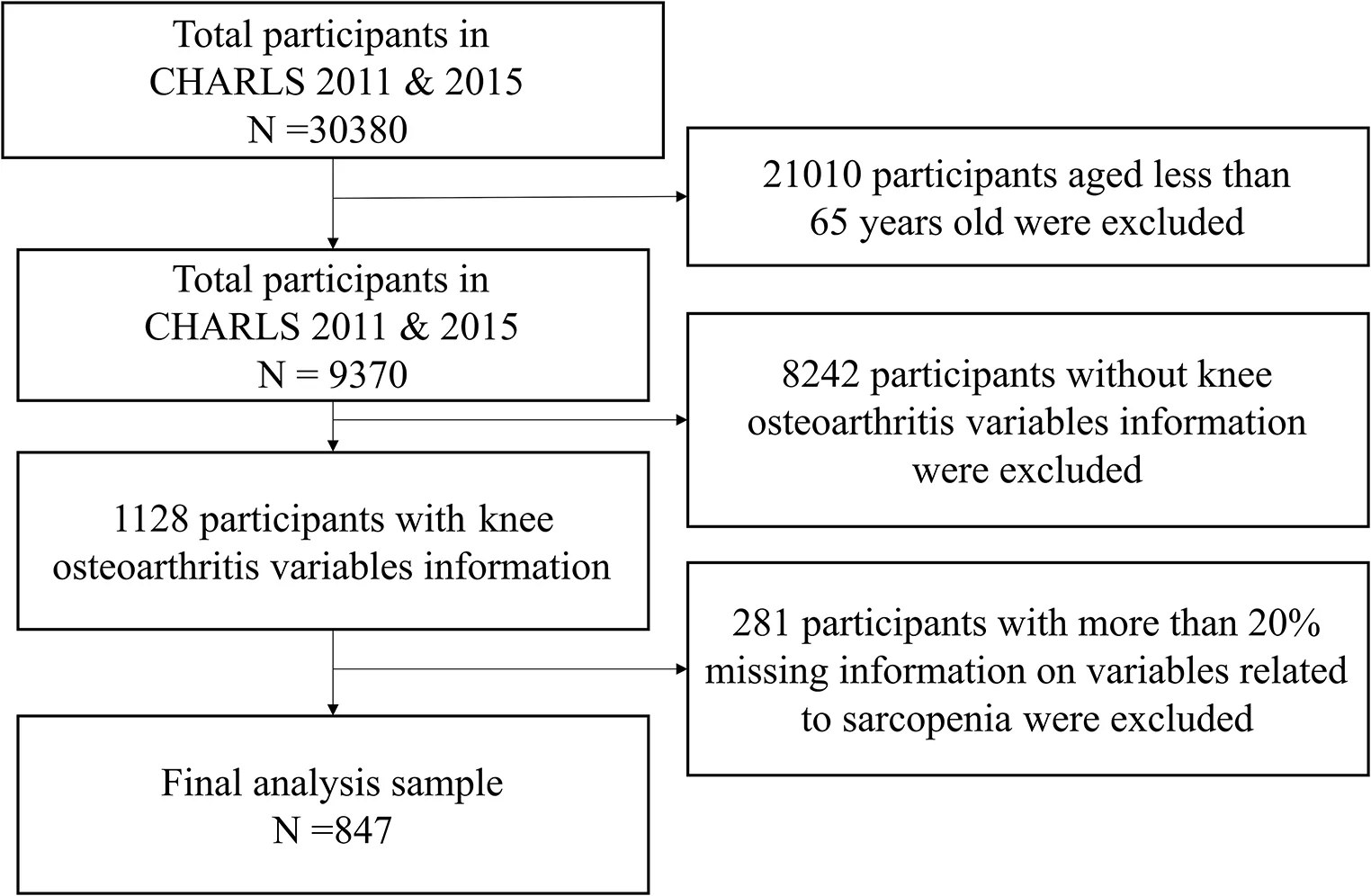
Sample screening

We utilized data from the China Health and Retirement Longitudinal Study (CHARLS), a nationwide survey targeting middle-aged and older Chinese adults. The CHARLS national baseline survey was conducted between June 2011 and March 2012, employing a four-stage stratified cluster sampling strategy across 28 provinces and municipalities in China, with follow-up assessments every two to three years thereafter [19]. The study was approved by the Institutional Review Board of Peking University (Approval No. IRB00001052–11015), and all participants provided informed consent. All procedures adhered to the guidelines of the Declaration of Helsinki and its subsequent amendments or equivalent ethical standards. In this study, owing to the inclusion of blood sample variables, we employed data from both the baseline survey (CHARLS 2011) and the follow-up survey (CHARLS 2015). These datasets include variables related to demographic characteristics, health status and functioning, lifestyle factors, and biomedical outcomes, which are all collected via structured questionnaires. After excluding participants with more than 20% missing data on relevant variables, a total of 847 individuals aged 65 and older were ultimately included. The process of sample screening is shown in Fig. 2.

### Assessment of sarcopenia in KOA patients

In this study, the incidence of sarcopenia in the population with symptomatic KOA was considered the outcome variable, with sarcopenia assessed according to the AWGS 2019 criteria, which consists of three components: muscle mass, muscle strength, and physical performance [5].

1) Muscle strength was assessed by measuring participants’ handgrip strength following the standard testing protocol. Low handgrip strength was defined as a maximum grip strength of less than 28 kg for men and less than 18 kg for women.

2) Muscle mass was assessed by estimating appendicular skeletal muscle mass (ASM) via anthropometric equations previously validated in the Chinese population [20]. Research has demonstrated a high degree of consistency between the appendicular skeletal muscle (ASM) equation and dual-energy X-ray absorptiometry (DXA). The equation model achieved an adjusted R² value of 0.90 and an estimated standard error of 1.63 kg [20, 21]. The critical value for reduced muscle mass was determined on the basis of sex-specific minimum adjustments of 20% for height-adjusted muscle mass (SMI) = ASM/Ht², with an ASM/Ht² value of < 5.26 kg/m² for females and < 7.0 kg/m² for males, which was defined as low muscle mass [20–22]. The ASM equation used was as follows:

1 $$\eqalign{ ASM = & 0.193 \times Weight\left( {kg} \right) + 0.107 \times Height\left( {cm} \right) \cr & - 4.157 \times sex - 0.037 \times age\left( {years} \right) - 2.631 \cr} $$

As part of our study, participants’ height was measured with a stadiometer with 0.1 cm accuracy. The weight was determined to be accurate to 0.1 kg via a digital standing scale. The weight and height of individuals are measured in kilograms and centimeters, respectively. Gender is coded as 1 for male participants and 2 for female participants, and age is given in years.

3) Physical performance was evaluated via the five-time chair stand test. Participants who required 12 s or more to complete the test or were unable to finish it were classified as having poor physical performance [23].

Additionally, symptomatic KOA is defined, following the definitions used in previous studies [7], as physician-diagnosed arthritis combined with self-reported knee pain. The participant is classified as having symptomatic KOA if they answer ‘yes’ to the first two questions and ‘knee’ to the third. (1) Have you been diagnosed with arthritis or rheumatism by a doctor? (2) Do you often feel pain in your body? If the participant answers ‘yes’ to the second question, he/she will be asked (3) Which part of your body is in pain? Those who answer ‘knee’ to this question will be classified as symptomatic KOA patients.

On the basis of the objectives of this study, participants with symptomatic KOA were ultimately categorized into two groups according to the three components of muscle wasting status: ‘nonsarcopenia’ and ‘sarcopenia’. ‘Nonsarcopenia’ was defined as the absence of muscle weakness, low muscle mass, and reduced physical performance. Sarcopenia was defined as low muscle mass and muscle weakness, with or without decreased physical function.

### Predictor variables and dataset creation

Considering previous relevant studies and the principle that missing values for predictor variables do not exceed 20% 24, our study initially included 95 predictor variables, and we divided predictor variables into five parts: demographics, behavioral factors, health status and functioning, and physical examination indicators. All these variables can be acquired directly from the CHARLS questionnaire.

Seven demographic variables were included in the analysis: age, sex, education, marital status, household hukou, current residence, and monthly per capita consumption. Gender was categorized as male or female. Educational level was classified into five categories: illiterate, primary school, middle school, high school, and college or above. Marital status was divided into ‘married’ and ‘unmarried,’ where ‘married’ referred to participants who were currently married and living with their spouse, and ‘unmarried’ encompassed those who were separated, divorced, widowed, or never married. Household Hukou was categorized as agricultural or nonagricultural, current residence was categorized as rural or urban, and monthly per capita expenditure was log-transformed. With respect to behavioral factors, the study included past smoking status, past drinking status, average sleep duration per night, nap duration, participation in social activities, difficulties with eight types of activities (e.g., jogging, sitting up, stretching upward), and activities of daily living (ADL) status. Additionally, cooking fuel was included as a predictor variable on the basis of relevant research [25]. The average sleep duration per night and nap duration were treated as continuous variables, whereas the other behavioral factors were categorical. Specifically, all variables, except for cooking fuel, are classified as either ‘yes’ or ‘no’.

For health status and functioning, we referred to relevant research [14] and selected cognition, depression, physical disability, neurological injury (such as brain injury), sensory deficits (such as blindness, deafness, and muteness), and 13 commonly prevalent chronic diseases. In addition to the arthritis studied in this research, hypertension, dyslipidemia, diabetes, cancer, and diseases affecting organs such as the lungs, liver, heart, and kidneys were also included. Additionally, other factors, such as a history of asthma, pain, surgeries (e.g., cataracts or hip fractures), the use of assistive devices such as hearing aids, and health indicators such as tooth loss, were assessed. Furthermore, we assessed variables related to accidents, such as falls, vision and hearing impairments, and self-rated health assessments. Specifically, hearing and self-rated health are categorized as ‘excellent,’ ‘very good,’ ‘good,’ ‘fair,’ or ‘poor,’ whereas the other variables are classified as ‘yes’ or ‘no.’

In the CHARLS questionnaire, depression is measured via the 10-item short version of the Center for Epidemiologic Studies Depression Scale (CES-D). The respondents are asked to rate eight negative statements (e.g., ‘I feel depressed’) and two positive statements (e.g., ‘I feel happy’) on a four-point scale. We reverse-scored the two positive statements and summed the scores of the ten items. On the basis of the findings of Roberts and colleagues [26], a CES-D score ≥ 16 is considered indicative of depressive symptoms, whereas a score < 16 indicates no depressive symptoms.

Cognition in CHARLS is assessed through three components: the Telephone Interview for Cognitive Status (TICS), word recall, and figure drawing, with higher scores indicating better cognitive function. In the TICS section, participants earn 1 point for correctly naming the year, month, day, week, and season, as well as 1 point for each correct subtraction of 7, up to five times, resulting in a total score of 0–10. The word recall task involves presenting participants with 10 words, which they attempt to recall within a short period; each correctly recalled word is given a score of 1 point, and the average score from two trials is calculated. In the figure drawing task, participants are asked to replicate a shape on a blank sheet of paper, earning 1 point for accurate reproduction. The overall cognition score ranges from 0 to 21 points.

For the physical examination indicators, we included 10 basic examination variables: BMI, upper arm length, knee height, waist circumference, respiratory measurements, systolic blood pressure, diastolic blood pressure, pulse, bilateral foot length (anterior‒posterior), and standing measurements of both feet (anterior‒posterior). Blood laboratory biochemical indicators, including 16 blood test parameters, such as leukocytes and hemoglobin, were also considered. After completing the collection of the dataset’s predictor variables, multiple imputations were performed for missing values.

During the variable selection process, the calculation of the ASM inherently incorporates key factors such as weight, height, sex, and age. Given that ASM is a pivotal indicator for diagnosing sarcopenia and that these factors are intrinsically associated with the condition, their inclusion in the predictive model could result in an overemphasis on these variables, thereby introducing multicollinearity and redundancy. To prevent such issues and ensure the independence and precision of the prediction model, these variables were subsequently excluded from the selection process.

### Feature selection via LASSO and logistic regression

In this study, we utilized data from CHARLS 2011 and CHARLS 2015 for analysis. For binary classification predictor variables, chi-square tests were employed; for continuous variables, independent samples t tests were conducted; and for ordinal categorical variables, Mann‒Whitney U tests were applied. We subsequently employed least absolute shrinkage and selection operator (LASSO) regression analysis to construct and validate the model. This approach effectively manages high-dimensional datasets with multicollinearity, facilitates variable selection, and enhances model interpretability. Compared with rigid networks and elastic net models, LASSO offers greater flexibility in variable selection and sparsity, making it more suitable for our research objectives and dataset characteristics, thereby improving the model’s accuracy and conciseness.

The tuning parameter lambda for LASSO regression analysis was determined through 10-fold cross-validation, and the most significant features were selected via the LASSO algorithm. Finally, the selected predictors were included in a binary logistic regression analysis, with predictors with a P value less than 0.05 included in the variable selection.

### Model development and comparison

The filtered dataset was divided into two subsets at a 7:3 ratio (training-val set and testing set). The training-val set was used for model training and cross-validation to comprehensively evaluate the model’s overall performance and stability and to prevent both overfitting and underfitting. The test set was independently partitioned at the beginning for the final performance evaluation, assessing the model’s generalization ability when encountering unseen data. There was no overlap between the two datasets. The prediction model was developed using the features after selection. Eight machine learning models—K-nearest neighbors (KNN), naive Bayes, random forest (RF), support vector machine (SVM), multilayer perceptron (MLP), extreme gradient boosting (XGBoost), light gradient boosting machine (LGBM), and categorical gradient boosting (CatBoost)—were used to predict whether symptomatic KOA patients are affected by sarcopenia.

As a result of using the training-val set, we built, trained, and validated the model via 5-fold cross-validation to provide effective verification of the model’s reliability and stability while preventing overfitting and underfitting. The accuracy, precision, recall/sensitivity, specificity, and F1 score are reported as the mean and standard deviation. The mean and variance of these metrics provide both a comprehensive evaluation of the model’s performance and insights into its variability, helping to reveal the model’s stability. For each fold, we also plotted the receiver operating characteristic (ROC) curve, which illustrates the trade-off between false positives and true positives in the model’s classification. After the completion of model construction, we utilized a full training-val set for training and conducted testing on the test set, which the model had never encountered, to validate the model’s generalization performance on unseen data. A wide range of performance metrics were reported from the test results, as were the ROC curve, calibration curve, and decision curve analysis (DCA) curve. The curves not only demonstrated the accuracy and consistency of the model’s predicted probabilities but also provided valuable insights and scientific evidence for clinical decision-making by assessing the model’s clinical utility across various thresholds.

### Model explanation based on SHAP

Interpreting machine learning models accurately presents a significant challenge. SHAP (SHapley Additive exPlanation) is a method designed to elucidate machine learning model predictions. It is grounded in the concept of Shapley values, a fair approach from game theory used to allocate cooperative gains among participants. SHAP values quantify the contribution of each feature to the prediction outcome, thereby enhancing the understanding of the model’s decision-making process. This implementation aims to address the ‘black box’ problem in machine learning.

### Web application based on Streamlit

To increase the practicality of the model in clinical settings, the final prediction model was implemented in a web application built on the Streamlit framework. When the values of the corresponding features in the final model are provided, the application can return the probability of symptomatic KOA patients concurrently having sarcopenia, along with a force plot for individual features.

## Results

### Participants characteristics

This study included a total of 847 elderly individuals (aged 65 years and above). Owing to space limitations, Table 1 presents the baseline characteristics of the variables selected through LASSO for the study population, while the complete baseline information for all predictive variables can be found in the *supplementary materials.* Among the participants, 301 were male (35.54%), and 546 were female (64.46%). The prevalence of sarcopenia among symptomatic KOA patients was 39.91%.

**Table 1:** Baseline characteristics of the study population (variables selected by LASSO)

Variable	Non-Sarcopenia (*n* = 509)	Sarcopenia (*n* = 338)	*P* value
Upper Arm Length (cm)	33.29 ± 2.37	32.91 ± 3.83	0.078
Waist Circumference (cm)	88.65 ± 15.25	75.24 ± 13.37	< 0.001
Ability to Stand on One Leg for 10 s			0.884
Yes	486(95.5%)	322(95.3%)	
No	23(4.5%)	16(4.7%)	
BMI (kg/m^2^)	25.39 ± 3.54	19.76 ± 1.93	< 0.001
Hemoglobin (g/dL)	13.70 ± 1.99	13.11 ± 1.68	< 0.001
Red Blood Cell Hematocrit (%)	41.26 ± 5.68	39.33 ± 5.61	< 0.001
Urea Nitrogen (mg/dL)	16.69 ± 4.90	16.54 ± 5.24	0.662
Low-Density Lipoprotein (mg/dL)	111.29 ± 33.04	105.97 ± 33.75	0.023
Total Cholesterol (mg/dL)	193.26 ± 37.72	186.71 ± 40.93	0.017
Uric Acid (mg/dL)	4.97 ± 1.41	4.58 ± 1.35	< 0.001
Cystatin C (mg/L)	1.00 ± 0.26	1.04 ± 0.29	0.046
Education Level			0.114
Illiterate	220(43.2%)	160(47.3%)	
Primary school	244(47.9%)	154(45.6%)	
Junior high school	40(7.9%)	18(5.3%)	
High school	5(1.0%)	3(0.9%)	
College and above	0(0%)	3(0.9%)	
Marital Status			0.011
Never married	132(25.9%)	115(34.0%)	
Married	377(74.1%)	223(66.0%)	
Hukou			< 0.001
Agricultural	444(87.2%)	322(95.3%)	
Non-agricultural	65(12.8%)	16(4.7%)	
History of Falls			0.919
Yes	191(37.5%)	128(37.9%)	
No	318(62.5%)	210(62.1%)	
Hearing Level			0.068
Very Good	24(4.71%)	14(4.14%)	
Good	54(10.61%)	35(10.36%)	
Fair	269(52.85%)	157(46.45%)	
Poor	162(31.83%)	132(39.05%)	
Average Nightly Sleep Duration (h)	5.49 ± 2.33	5.33 ± 2.44	0.350
At least 10 min of Moderate Physical Activity			0.146
Yes	137(26.9%)	76(22.5%)	
No	372(73.1%)	262(77.5%)	
Muteness or Severe Speech Impairment			0.558
Yes	4(0.8%)	4(1.2%)	
No	505(99.2%)	334(98.8%)	
Hypertension			< 0.001
Yes	239 (47.0%)	95 (28.1%)	
No	270 (53.0%)	243 (71.9%)	
Dyslipidemia			< 0.001
Yes	89(17.5%)	16(4.7%)	
No	420(82.5%)	322(95.3%)	
Cancer			0.219
Yes	12(2.4%)	4(1.2%)	
No	497(97.6%)	334(98.8%)	
Chronic Lung Disease			0.209
Yes	112(22.0%)	87(25.7%)	
No	397(78.0%)	251(74.3%)	
Liver Disease			0.492
Yes	46(9.0%)	26(7.7%)	
No	463(91.0%)	312(92.3%)	
Heart Disease			< 0.001
Yes	133(26.1%)	55(16.3%)	
No	376(73.9%)	283(83.7%)	
Stomach Diseases			0.299
Yes	218(42.8%)	157(46.4%)	
No	291(57.2%)	181(53.6%)	
Cataract Surgery			0.656
Yes	26(5.1%)	15(4.4%)	
No	483(94.9%)	323(95.6%)	
Glaucoma			0.993
Yes	12(2.4%)	8(2.4%)	
No	497(97.6%)	330(97.6%)	
Complete Tooth Loss			< 0.01
Yes	103(20.2%)	99(29.3%)	
No	406(79.8%)	239(70.7%)	
Headaches			0.253
Yes	284(55.8%)	202(59.8%)	
No	225(44.2%)	136(40.2%)	
Shoulder Pain			0.965
Yes	314(61.7%)	208(61.5%)	
No	195(38.3%)	130(38.5%)	
Finger Pain			0.339
Yes	209(41.1%)	150(44.4%)	
No	300(58.9%)	188(55.6%)	
Waist Pain			0.522
Yes	374(73.5%)	255(75.4%)	
No	135(26.5%)	83(24.6%)	
Neck Pain			0.501
Yes	211 (41.5%)	148 (43.8%)	
No	298 (58.5%)	190 (56.2%)	
Participation in Social Activities			0.121
Yes	246(48.3%)	145(42.9%)	
No	263(51.7%)	193(57.1%)	
History of Alcohol Consumption			0.615
Yes	214(42.0%)	148(43.8%)	
No	295(58.0%)	190(56.2%)	
Difficulty Climbing Stairs			0.160
Yes	402(79.0%)	253(74.9%)	
No	107(21.0%)	85(25.1%)	
Difficulty Reaching Upward			0.118
Yes	156(30.6%)	121(35.8%)	
No	353(69.4%)	217(64.2%)	
ADL			0.902
Yes	213(41.8%)	140(41.4%)	
No	296(58.2%)	198(58.6%)	
Primary Cooking Fuel			< 0.01
Coal	50(9.82%)	19(5.62%)	
Natural Gas	45(8.84%)	22(6.51%)	
Marsh Gas	5(0.98%)	7(2.07%)	
Liquefied Petroleum Gas	47(9.23%)	15(4.44%)	
Electric	72(14.15%)	53(15.68%)	
Crop Residue/Wood Burning	285(55.99%)	220(65.09%)	
Other	5(0.98%)	2(0.59%)	
Self-Rated Health			0.956
Very Good	2(0.39%)	2(0.59%)	
Good	14(2.75%)	8(2.37%)	
Fair	139(27.31%)	93(27.51%)	
Poor	277(54.42%)	185(54.74%)	
Very poor	77(15.13%)	50(14.79%)	
Depression			< 0.01
Yes	374(73.5%)	275(81.4%)	
No	135(26.5%)	63(18.6%)	

### Feature selection via LASSO and logistic regression

**Fig. 3:**
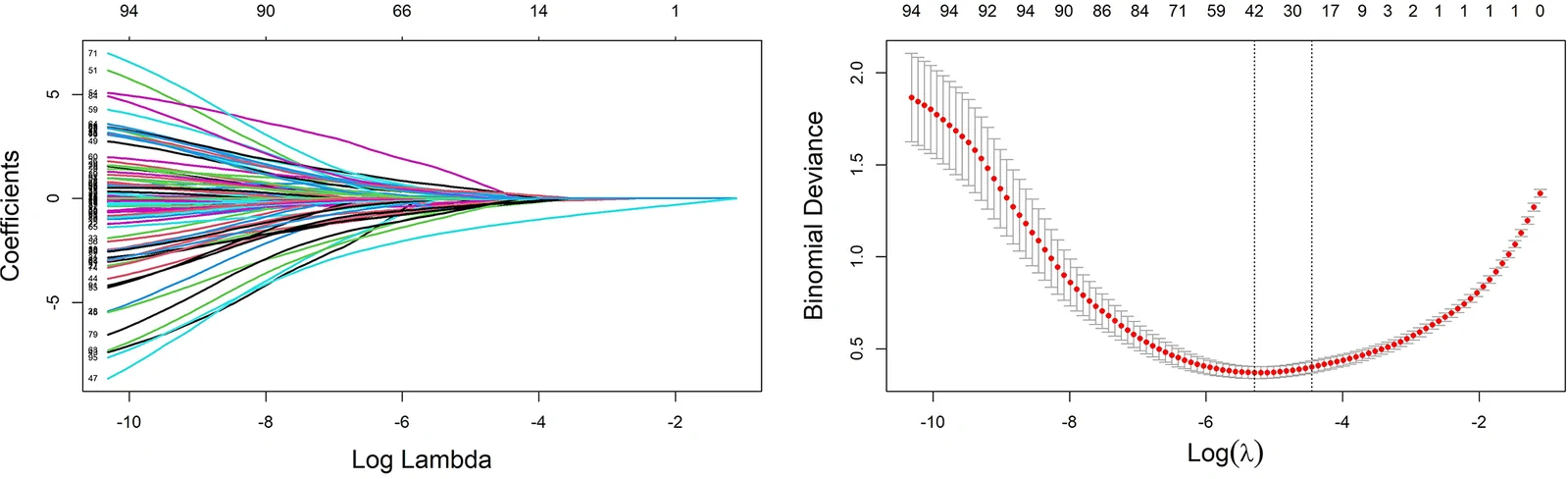
**Left** LASSO Path Plot: Regression coefficients decrease toward zero as log lambda increases, indicating that stronger regularization increases. This demonstrates the model’s simplification by reducing active predictors with increasing penalty strength. **Right** LASSO Selection Path Plot: Vertical dashed lines highlight the log(λ) for minimum error (lambda.min) on the left and the log(λ) at one standard error from the minimum (lambda.1se) on the right. Binomial deviance shows the model’s loss function calculated during cross-validation for each fold

As lambda decreases in the LASSO path plot (Fig. 3** Left**), the number of predictive variables increases, illustrating the trade-off between model complexity and predictive capability. The cross-validation error associated with each lambda value was also examined to determine the optimal lambda value (Fig. 3** Right**). In our study, lambda.min was selected because it minimizes the cross-validation error, ensuring that all significant predictor variables are included. As a result of this choice, the predictive accuracy of the model becomes improved through the capture of complex relationships within the data. Even though lambda.min may contain more variables, it optimizes the model’s performance and robustness, which results in improved overall predictive performance.

We selected 42 predictive variables on the basis of lambda.min, as shown in Fig. 3** (right)**. The variance inflation factors (VIFs) obtained from the covariance diagnostics were all less than 10, which indicates that there was no multicollinearity among these variables. Next, we included the 42 predictive variables in a binary logistic regression analysis. After 7 iterations, the Prob > chi2 value of our model was 0.000, which is less than 0.05, indicating that our model is statistically significant. The results are shown in Table 2. Finally, on the basis of the principle of *P* < 0.05, we selected 6 variables in Table 2: BMI, upper arm length (UAL), marital status (MS), total cholesterol (TC), cystatin C (CysC), and shoulder pain (SP).

**Table 2:** Results of variable selection based on binary logistic regression

Variables	OR (95%CI)	*P* value
BMI	0.124(0.085 ~ 0.183)	< 0.001
Upper Arm Length	0.740(0.669 ~ 0.819)	< 0.001
Marital Status	0.300(0.133 ~ 0.675)	0.004
Total Cholesterol	1.027(1.004 ~ 1.049)	0.020
Cystatin C	4.836(1.167 ~ 20.039)	0.030
Shoulder Pain	0.337(0.146 ~ 0.777)	0.011

### Model development and comparison

**Fig. 4:**
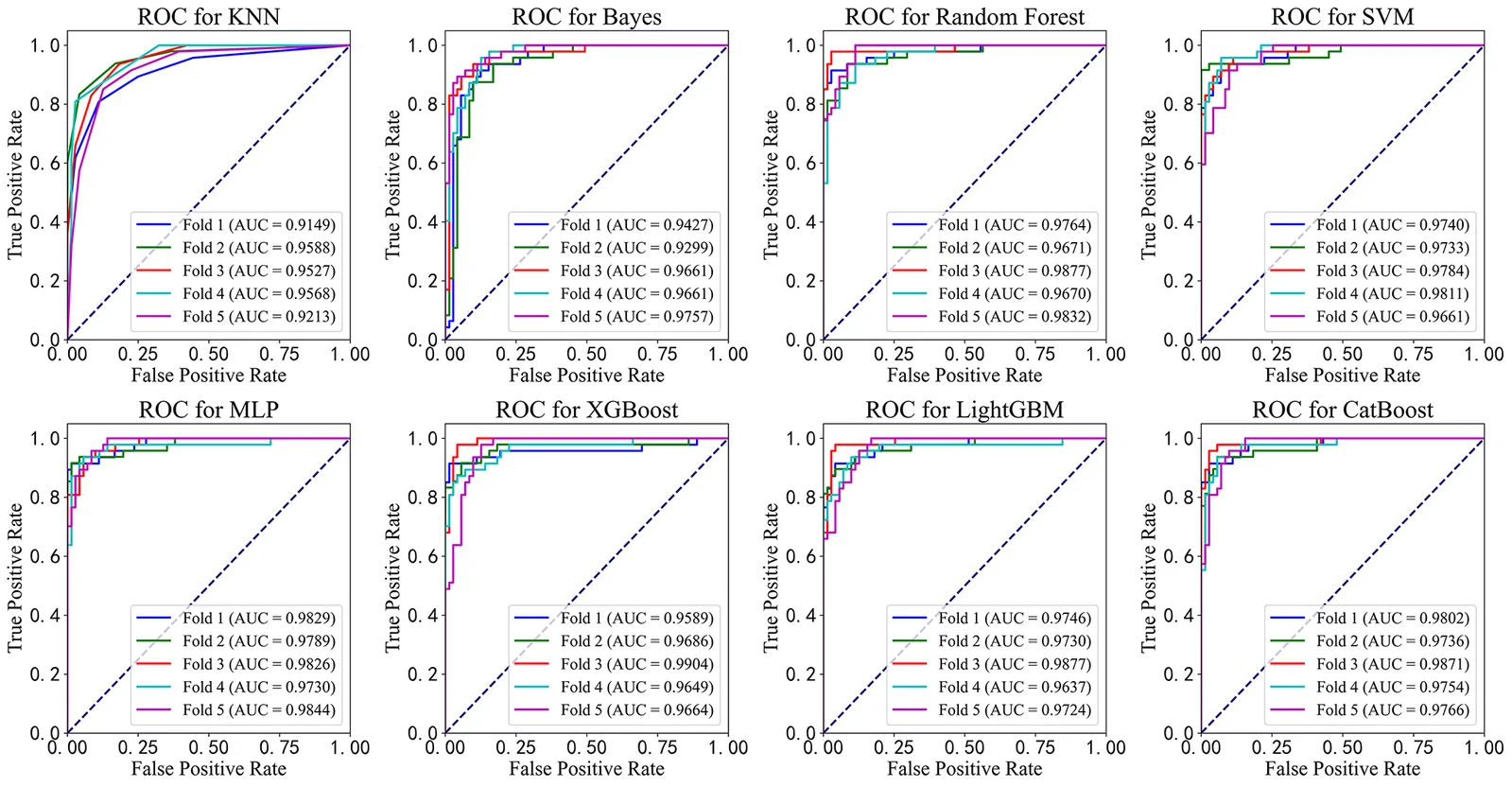
ROC curves for eight machine learning models during 5-fold cross-validation on the training-val set

**Fig. 5:**
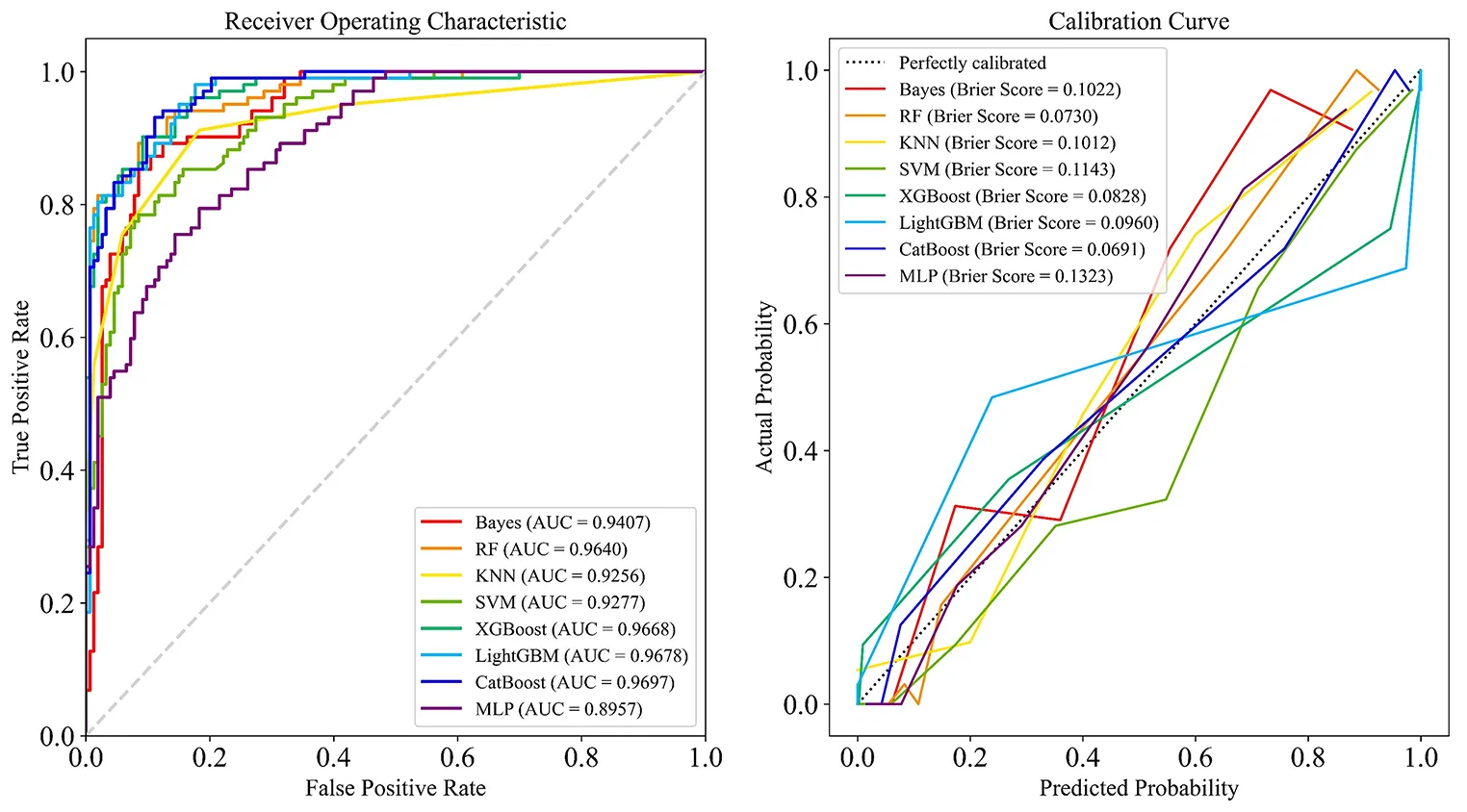
ROC curves and calibration curves for 8 models on the independent test set from the final test. **Left** ROC curve of the independent test set: ROC curves for eight different models, highlighting their ability to distinguish between classes. The AUC quantifies the model’s accuracy, with higher values indicating superior performance. **Right** Calibration Curves on the independent test set: Calibration curves for the models, comparing the accuracy of the predicted probabilities against the actual outcomes. A calibration curve that closely follows the perfectly calibrated curve indicates greater reliability, indicating that the model’s predictions align well with actual outcomes and are more trustworthy in real-world scenarios. Each model’s Brier score is noted, with lower scores reflecting more precise probability predictions, thus indicating the reliability of the model in practical scenarios

We first developed eight machine learning models using the training-val set and employed 5-fold cross-validation to comprehensively assess their performance and stability. The results are presented in Table 3, and the ROC curves for each fold are shown in Fig. 4. To ensure the model’s generalizability and prevent data leakage, we reserved an independent test set at the outset for the final performance evaluation. This approach allowed us to assess the model’s ability to generalize to unseen data. We then conducted the final performance testing of the eight models on the test set and plotted the ROC curves for each model’s predictions, as shown in Table 4; Fig. 5.

**Table 3:** Results from 5-fold cross-validation on the validation set

Model	Accuracy	Precision	Recall/Sensitivity	Specificity	F1	AUC
KNN	0.8834 ± 0.0234	0.8779 ± 0.0603	0.8262 ± 0.0181	0.9214 ± 0.0426	0.8502 ± 0.0263	0.9409 ± 0.0210
Bayes	0.9004 ± 0.0273	0.8990 ± 0.0463	0.8477 ± 0.0539	0.9355 ± 0.0334	0.8714 ± 0.0357	0.9561 ± 0.0191
RF	0.9172 ± 0.0324	0.9443 ± 0.0338	0.8434 ± 0.0772	0.9662 ± 0.0214	0.8892 ± 0.0455	0.9763 ± 0.0093
SVM	0.9189 ± 0.0245	0.9364 ± 0.0379	0.8557 ± 0.0556	0.9607 ± 0.0251	0.8931 ± 0.0344	0.9746 ± 0.0057
MLP	0.9307 ± 0.0111	0.9236 ± 0.0338	0.9025 ± 0.0325	0.9495 ± 0.0234	0.9121 ± 0.0142	0.9804 ± 0.0046
XGBoost	0.9324 ± 0.0262	0.9189 ± 0.0501	0.9152 ± 0.0656	0.9437 ± 0.0411	0.9149 ± 0.0338	0.9699 ± 0.0120
LGBM	0.9240 ± 0.0306	0.9257 ± 0.0412	0.8813 ± 0.0614	0.9522 ± 0.0292	0.9018 ± 0.0406	0.9743 ± 0.0086
CatBoost	0.9324 ± 0.0261	0.9369 ± 0.0424	0.8941 ± 0.0794	0.9578 ± 0.0331	0.9123 ± 0.0377	0.9786 ± 0.0053

We generated a calibration plot for the prediction results of each model on the test set and calculated the Brier score for each, as shown in Fig. 5** (right)**. Calibration plots are used to assess the accuracy of prediction models by illustrating the relationship between predicted event probabilities and actual occurrence probabilities. Ideally, the calibration curve should closely follow the diagonal line in the figure, indicating that the predicted probabilities align well with the actual occurrence probabilities.

Among the eight models, CatBoost, XGBoost, and MLP demonstrated similar performances during cross-validation. However, in the independent test set, the MLP model exhibited significantly weaker overall performance and poorer generalizability. Although XGBoost achieved classification metrics comparable to those of CatBoost on the test set, its Brier score and calibration curve were inferior, indicating less reliable probability predictions. In contrast, CatBoost demonstrated superior performance in both calibration analyses and probability estimates, reflecting more accurate and dependable predictions. Consequently, considering both classification performance and probability calibration, CatBoost was selected as the final model for this study.

**Table 4:** Final testing results on the independent test set

Model	Accuracy	Precision	Recall/Sensitivity	Specificity	F1	AUC	Brier Score
KNN	0.8667	0.8953	0.7549	0.9412	0.8191	0.9256	0.1012
Bayes	0.8627	0.8941	0.7451	0.9412	0.8128	0.9407	0.1022
RF	0.8941	0.9032	0.8235	0.9412	0.8615	0.9640	0.0730
SVM	0.8157	0.7311	0.8529	0.7908	0.7873	0.9277	0.1143
MLP	0.8039	0.8171	0.6569	0.9020	0.7283	0.8957	0.1323
XGBoost	0.8941	0.8641	0.8725	0.9085	0.8683	0.9668	0.0828
LGBM	0.8863	0.8544	0.8627	0.9020	0.8585	0.9678	0.0960
CatBoost	0.8902	0.8627	0.8627	0.9085	0.8627	0.9697	0.0691

**Fig. 6:**
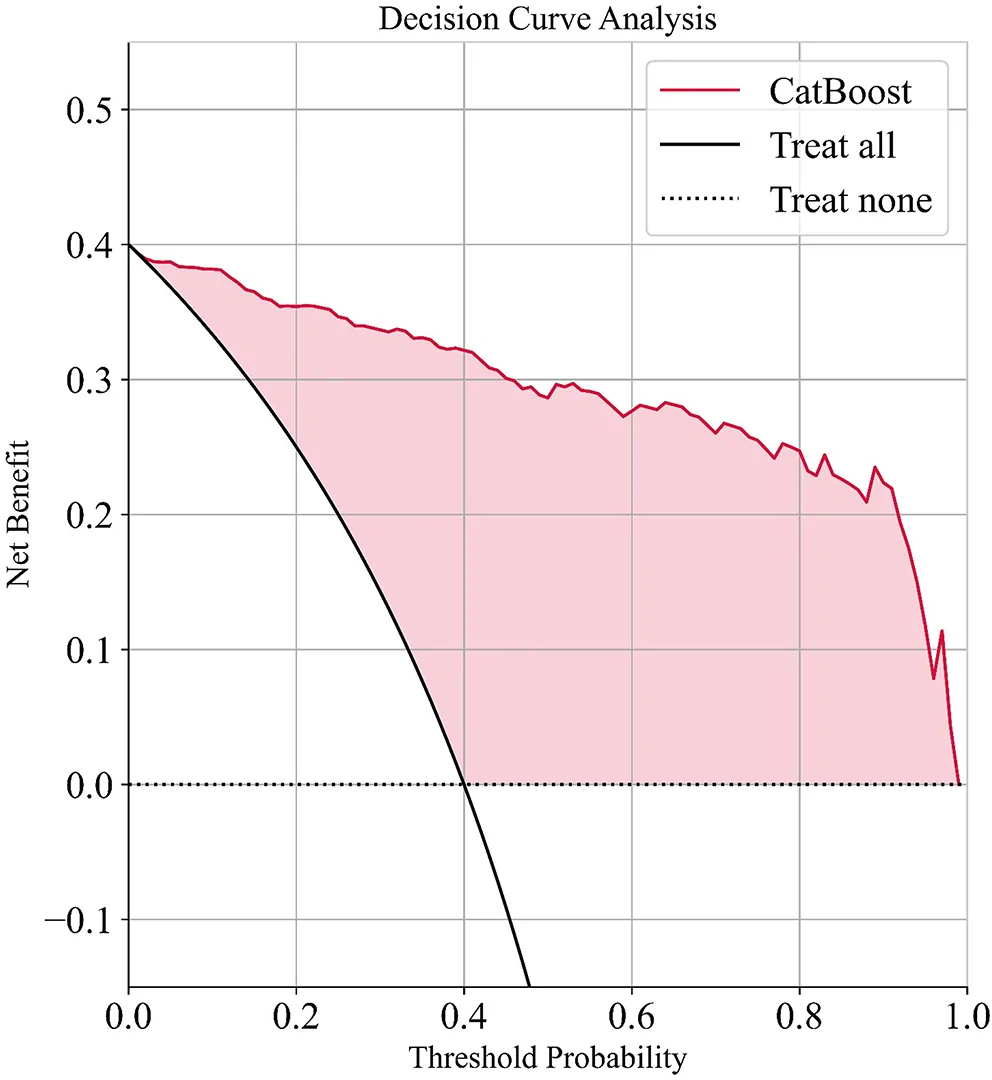
DCA curve for the CatBoost algorithm on the independent test set from the final test. The DCA curve depicting the net benefits of various decision-making strategies at different probability thresholds. The x-axis represents the probability thresholds, and the y-axis shows the corresponding net benefits. The curve illustrates the trade-offs between benefits and harms for the ‘treat all’, ‘treat none’, and model-based strategies, highlighting the clinical utility of the predictive model in decision-making

Decision curve analysis (DCA) is a widely used method to assess clinical utility. We plotted the DCA curve for the optimal model, CatBoost, on the basis of the test set results. In the DCA, ‘treat all’ means that all patients receive treatment, whereas ‘treat none’ means that no patients receive treatment. As shown in Fig. 6, our predictive model delivers greater net benefits across various threshold probabilities, enhancing clinical decision-making and improving patient outcomes.

### Model explanation

Since it is challenging for clinicians to accept predictions from models that cannot be directly interpreted, we used the SHAP method to explain the model’s output by calculating each variable’s contribution to the predictions. This interpretable method provides two types of explanations: global explanations at the feature level and local explanations at the individual level.

**Fig. 7:**
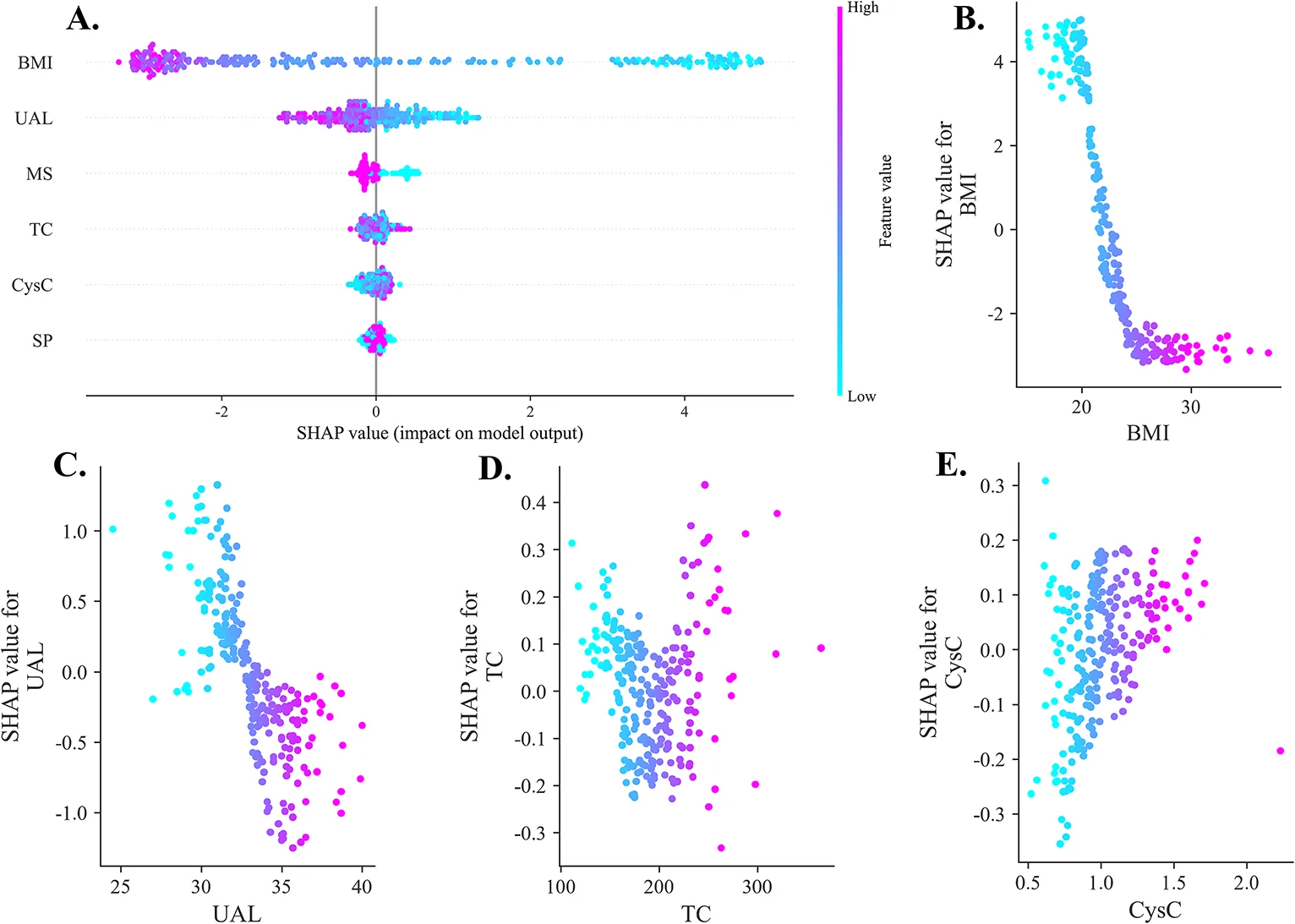
(**A**) SHAP summary dot plot: SHAP values of features correlated with an increased risk of sarcopenia in KOA patients. Each patient is represented by a single dot per feature in the model, which is positioned according to their SHAP value. The color coding of the dots reflects the actual values of the features, where purple indicates higher values and blue indicates lower values. (**B**-**E**) SHAP dependence plot. Each dependence plot shows how a single feature affects the output of the prediction model, and each dot represents a single patient. For example, both low BMI and UAL values significantly increase SHAP values, indicating a higher risk of classification as ‘sarcopenia,’ whereas higher BMI and UAL values significantly decrease SHAP values, suggesting a lower risk. Additionally, elevated CysC levels, an unmarried status, and extremely high or low TC levels significantly increased SHAP values, further indicating an elevated risk of sarcopenia. SHAP values are represented on the y-axis, whereas the actual values of these features are on the x-axis

Global explanations describe the overall functionality of the model. As shown in the SHAP summary plot (Fig. 7. **A**) and dependence plots (Fig. 7. **B-E**), the average SHAP values are used to evaluate the contribution of features to the model, displayed in descending order. The SHAP summary plot illustrates how SHAP values of features correlate with the increased risk of sarcopenia in KOA patients, where each patient is represented by a single dot per feature positioned according to their SHAP value. The color coding of the dots reflects the actual values of the features, with purple indicating higher values and blue indicating lower values. Furthermore, the dependence plots assist in elucidating how individual features impact the output of the prediction model, particularly facilitating the analysis of continuous variables by comparing their actual values with corresponding SHAP values. This comprehensive visualization allows for a clear interpretation of the relationships between key predictors and sarcopenia risk in symptomatic KOA patients.

### Convenient application for clinical utility

**Fig. 8:**
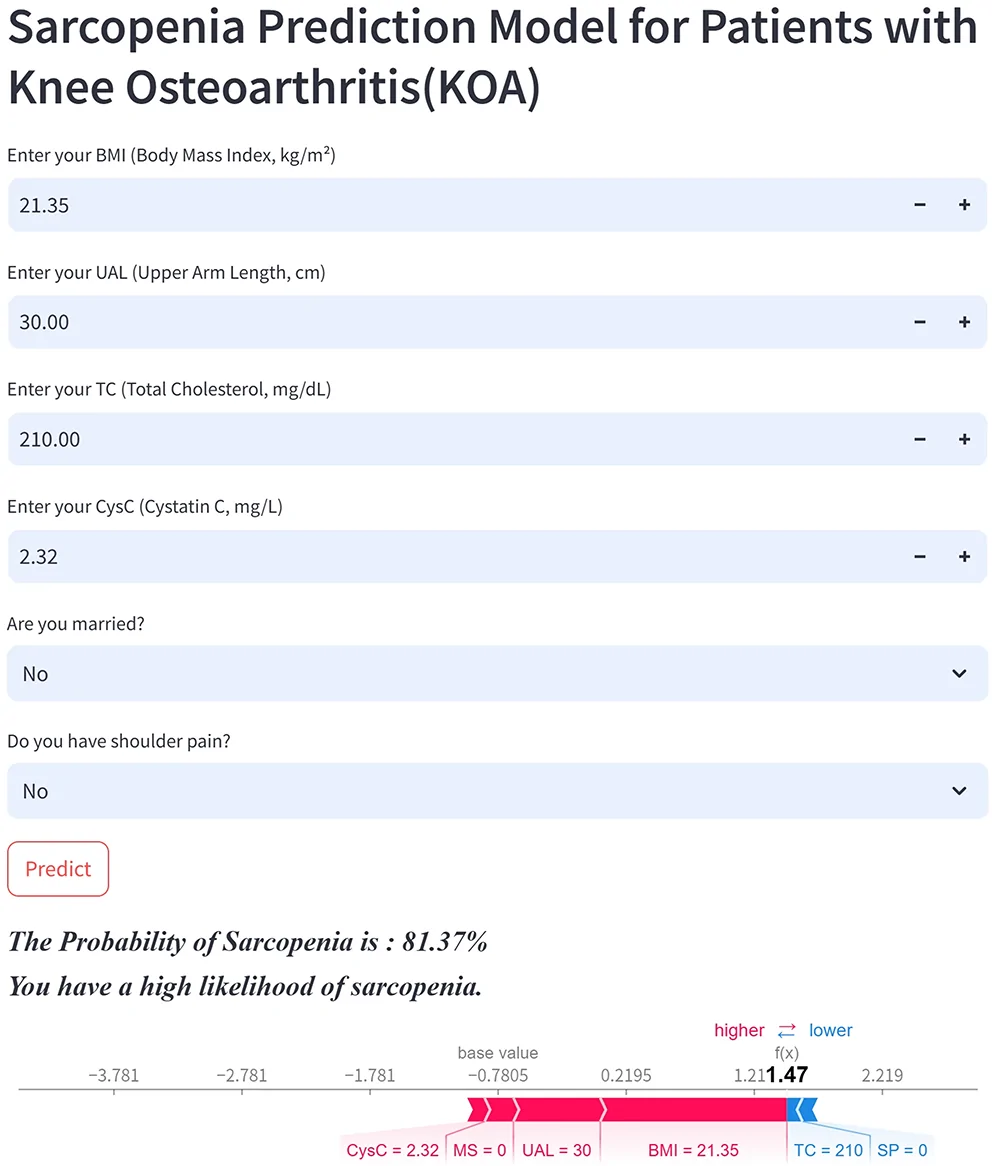
Convenient application for clinical utility. This user-friendly application uses the CatBoost model with nine features to predict sarcopenia in patients with KOA. Users can input actual values for these features, and the application immediately calculates the probability of sarcopenia, which is displayed here as 81.37%. Additionally, the force plot highlights the influence of each feature on the prediction outcome: blue features on the right suggest a lower probability of sarcopenia, whereas red features on the left increase the likelihood of this condition, clearly delineating the factors that drive the model’s predictions

The final prediction model was implemented in a web application to enhance its practicality in clinical settings, as shown in Fig. 8. When the actual values of the required features are input into the model, this application automatically predicts the probability of patients with KOA simultaneously having sarcopenia. Additionally, a force plot for individual subitems is displayed to indicate the decision-making process regarding features: the blue features on the right push the prediction toward ‘nonsarcopenia,’ whereas the red features on the left push the prediction toward ‘sarcopenia.’ This web application is accessible online at http://106.54.231.169/.

## Discussion

In this study, eight machine learning models were developed, evaluated, and compared for the prediction of sarcopenia risk among patients with symptomatic KOA via data from CHARLS. A comparison of the eight machine learning models evaluated shows that CatBoost demonstrates robust performance across classification and calibration metrics. Owing to its strong classification, Brier score and calibration curve performance, it is well suited for this application. Additionally, the model’s integration with SHAP (SHapley additive explanation) values, which enhances the interpretability of the model. As a result, CatBoost can be an invaluable tool for identifying sarcopenia risk in symptomatic KOA patients, allowing for the implementation of timely interventions to improve the health outcomes of patients.

According to our findings, sarcopenia is associated with low BMI. The reason could be that BMI has become a well-recognized measure of nutritional status on the basis of the relationship between weight and height. In addition to supporting this conclusion, previous studies have shown that malnutrition can cause muscle loss, weakness, and atrophy in individuals [1, 3, 27]. Furthermore, we found that a short UAL is associated with an increased risk of sarcopenia. A possible explanation is that UAL provides significant information concerning height [28, 29], which is a significant factor in determining the risk of developing sarcopenia. Consequently, a shorter UAL indicates a shorter height, which may affect the assessment of sarcopenia.

The results of our study indicate that unmarried individuals lose more muscle mass, whereas married individuals seem to be more protected from sarcopenia. The possibility exists that pain and mobility limitations could complicate daily activities within KOA, whereas married individuals could share family responsibilities and encourage each other to exercise regularly. However, unmarried individuals may experience limited social interaction and a lack of motivation to engage in physical activity, which may result in muscle atrophy. There is new evidence that supports our conclusion that marital status affects the social, behavioral, and nutritional environment of older adults, thereby affecting the risk of sarcopenia [30, 31]. Additionally, a systematic review is consistent with our findings, showing that unmarried individuals are almost twice as likely to experience frailty as married individuals [32].

On the basis of the results of our study, TC had a ‘V-shaped’ association with sarcopenia risk, in which individuals with both low and high TC levels were more likely to lose muscle mass. Low TC can be caused by inadequate nutritional intake or insufficient energy intake, thereby impairing muscle protein synthesis and repair. In contrast, elevated TC is often associated with chronic inflammation and metabolic dysfunction, further promoting muscle catabolism. Numerous studies have shown that lipid profiles play a critical role in the assessment of muscle health [33–35]. In terms of clinical intervention, maintaining TC within a healthy range could be a critical strategy to avoid sarcopenia and improve health.

Additionally, higher CysC levels were associated with a greater risk of sarcopenia in this study. This finding is consistent with previous findings that low Cr/CysC ratios can be a strong predictor of sarcopenia [36–40]; indeed, elevated CysC levels indicate a decrease in muscle mass because of a low Cr/CysC ratio. Elevated CysC may reflect an imbalance in muscle protein turnover. Patients with KOA are particularly vulnerable to catabolic processes that may result in muscle loss and functional decline. As a result, routine clinical assessments of CysC levels should be performed to identify individuals at risk of sarcopenia. We also found that shoulder pain (SP) is likely to contribute to sarcopenia with symptomatic KOA, and several studies have supported our conclusion that chronic shoulder disorders (e.g., rotator cuff tears) are associated with increased muscle loss and sarcopenia severity [41–43]. Nevertheless, as is evident from the SHAP summary plot, shoulder pain appears to have had a relatively limited impact on sarcopenia with symptomatic KOA. Although shoulder pain is recognized as a significant cause of disability and functional decline, it did not appear to have a significant effect on sarcopenia with KOA in our study population.

However, there are several limitations to our study. First, the cross-sectional design constrains our ability to infer causal relationships between predictive factors and sarcopenia risk. It is necessary to conduct longitudinal studies to establish causality and temporal associations. Second, CHARLS is a database focused on middle-aged and older adults. Since the focus of this study was on individuals aged 65 and above, only 847 participants were included in the study after screening. Owing to the lack of a sufficient sample size, our findings may not be generalizable. Third, some variables with missing data required imputation, which may have introduced bias into the results. Although multiple imputation techniques were employed, residual confounding cannot be eliminated. In future studies, data from additional databases could be combined to increase the robustness of the results, thereby allowing for larger and more diverse sample populations.

## Conclusion

From the perspective of public health and aging, we developed an interpretable and high-performing machine learning model based on routine clinical data to predict the risk of sarcopenia in patients with symptomatic knee osteoarthritis (KOA). By incorporating easily obtainable clinical and demographic variables, this model serves as a valuable tool for early screening and risk assessment. It can aid health departments and clinicians in the early identification and follow-up of high-risk individuals, facilitate timely interventions to prevent or mitigate sarcopenia, and ultimately improve the quality of life and health outcomes of elderly patients.

## Electronic supplementary material

Below is the link to the electronic supplementary material.

Supplementary Material 1

Supplementary Material 2

## Data Availability

The data used in this study are from the CHARLS, which is publicly available. Researchers can access and download the CHARLS data from the official website at https://charls.pku.edu.cn/.
