# Supplementary material for: An explainable machine learning-based prediction model for sarcopenia in elderly Chinese people with knee osteoarthritis
Source: Aging Clin Exp Res. 2025 Mar 7;37(1):67. doi: 10.1007/s40520-025-02931-x (PMC11889032; doi:10.1007/s40520-025-02931-x)
Supplement: Supplementary file 1 — Supplementary Material 1 [file 40520_2025_2931_MOESM1_ESM.docx]

**Baseline Characteristics of the Study Population (All Variables)**

| **Variable** | **Non-Sarcopenia**  **(n=509)** | **Sarcopenia**  **(n=338)** | **P value** |
| --- | --- | --- | --- |
| Age (years old) | 70.18±4.64 | 72.18±5.58 | <0.001 |
| Gender |  |  | 0.870 |
| Male | 182(35.8%) | 119(35.2%) |  |
| Female | 327(64.2%) | 219(64.8%) |  |
| BMI (kg/m^2^) | 25.39±3.54 | 19.76±1.93 | <0.001 |
| Education Level |  |  | 0.114 |
| Illiterate | 220(43.2%) | 160(47.3%) |  |
| Primary school | 244(47.9%) | 154(45.6%) |  |
| Junior high school | 40(7.9%) | 18(5.3%) |  |
| High school | 5(1.0%) | 3(0.9%) |  |
| College and above | 0(0%) | 3(0.9%) |  |
| Marital Status |  |  | 0.011 |
| Never married | 132(25.9%) | 115(34.0%) |  |
| Married | 377(74.1%) | 223(66.0%) |  |
| Hukou |  |  | <0.001 |
| Agricultural | 444(87.2%) | 322(95.3%) |  |
| Non-agricultural | 65(12.8%) | 16(4.7%) |  |
| Current Residence |  |  | 0.027 |
| Urban | 230(45.19%) | 179(52.96) |  |
| Rural | 279(54.81%) | 159(47.04) |  |
| Log Monthly Per Capita Consumption | 5.82±1.02 | 5.82±1.09 | 0.984 |
| Upper Arm Length (cm) | 33.29±2.37 | 32.91±3.83 | 0.078 |
| Knee Height (cm) | 47.21±3.64 | 46.03±3.29 | <0.001 |
| Waist Circumference (cm) | 88.65±15.25 | 75.24±13.37 | <0.001 |
| Respiratory Measurement (L/min) | 228.25±100.15 | 199.88±94.60 | <0.001 |
| Systolic Blood Pressure (mmHg) | 137.37±21.27 | 133.10±22.89 | <0.01 |
| Diastolic Blood Pressure (mmHg) | 75.85±10.95 | 73.12±11.76 | <0.001 |
| Pulse (bpm) | 73.10±10.99 | 74.40±12.07 | 0.106 |
| White Blood Cells (in thousands) | 6.33±2.05 | 6.01±1.97 | 0.023 |
| Hemoglobin (g/dL) | 13.70±1.99 | 13.11±1.68 | <0.001 |
| Red Blood Cell Hematocrit (%) | 41.26±5.68 | 39.33±5.61 | <0.001 |
| Mean Corpuscular Volume (fL) | 92.27±7.84 | 90.64±9.31 | <0.01 |
| Platelets (10^9^/L) | 203.10±72.35 | 207.70±85.62 | 0.400 |
| Triglycerides (mg/dL) | 154.22±107.49 | 111.78±73.64 | <0.001 |
| Creatinine (mg/dL) | 0.83±0.26 | 0.81±0.26 | 0.463 |
| Urea Nitrogen (mg/dL) | 16.69±4.90 | 16.54±5.24 | 0.662 |
| High-Density Lipoprotein (mg/dL) | 50.03±11.97 | 56.46±14.59 | <0.001 |
| Low-Density Lipoprotein (mg/dL) | 111.29±33.04 | 105.97±33.75 | 0.023 |
| Total Cholesterol (mg/dL) | 193.26±37.72 | 186.71±40.93 | 0.017 |
| Fasting Blood Glucose (mg/dL) | 106.75±30.17 | 103.72±31.85 | 0.162 |
| Uric Acid (mg/dL) | 4.97±1.41 | 4.58±1.35 | <0.001 |
| Cystatin C (mg/L) | 1.00±0.26 | 1.04±0.29 | 0.046 |
| C-Reactive Protein (mg/L) | 3.43±6.40 | 4.11±12.53 | 0.301 |
| Glycated Hemoglobin | 5.82±0.91 | 5.60±0.79 | <0.001 |
| Ability to Stand on One Leg for 10s |  |  | 0.884 |
| Yes | 486(95.5%) | 322(95.3%) |  |
| No | 23(4.5%) | 16(4.7%) |  |
| Ability to Stand in a Straight Line for 30 or 60s |  |  | 0.783 |
| Yes | 327(64.2%) | 214(35.8%) |  |
| No | 182(63.3%) | 124(36.7%) |  |
| Experience with Traffic Accidents or Major Unexpected Injury Treatment |  |  | 0.029 |
| Yes | 39(7.7%) | 41(12.1%) |  |
| No | 470(92.3%) | 297(87.9%) |  |
| History of Falls |  |  | 0.919 |
| Yes | 191(37.5%) | 128(37.9%) |  |
| No | 318(62.5%) | 210(62.1%) |  |
| Hip Fracture |  |  | 0.959 |
| Yes | 26(5.1%) | 17(5.0%) |  |
| No | 483(94.9%) | 321(95.0%) |  |
| Myopia |  |  | 0.026 |
| Yes | 143(28.1%) | 72(21.3%) |  |
| No | 366(71.9%) | 266(78.7%) |  |
| Hearing Level |  |  | 0.068 |
| Very Good | 24(4.71%) | 14(4.14%) |  |
| Good | 54(10.61%) | 35(10.36%) |  |
| Fair | 269(52.85%) | 157(46.45%） |  |
| Poor | 162(31.83%) | 132(39.05%) |  |
| Self-Rated Health in Youth |  |  | <0.01 |
| Excellent | 61(11.98%) | 31(9.17%) |  |
| Very Good | 185(36.35%) | 98(28.99%) |  |
| Good | 121(23.77%) | 82(24.26%) |  |
| Fair | 94(18.47%) | 85(25.15%) |  |
| Poor | 48(9.43%) | 42(12.43%) |  |
| Average Nightly Sleep Duration (h) | 5.49±2.33 | 5.33±2.44 | 0.350 |
| At least 10 Minutes of Vigorous Physical Activity |  |  | 0.896 |
| Yes | 80(15.7%) | 52(15.4%) |  |
| No | 429(84.3%) | 286(84.6%) |  |
| At least 10 Minutes of Moderate Physical Activity |  |  | 0.146 |
| Yes | 137(26.9%) | 76(22.5%) |  |
| No | 372(73.1%) | 262(77.5%) |  |
| At least 10 Minutes of Walking |  |  | 0.028 |
| Yes | 213(41.8%) | 116(34.3%) |  |
| No | 296(58.2%) | 222(65.2%) |  |
| Physical Disability |  |  | 0.097 |
| Yes | 69(13.6%) | 33(9.8%) |  |
| No | 440(86.4%) | 305(90.2%) |  |
| Brain Damage or Intellectual Deficit |  |  | 0.348 |
| Yes | 47(9.2%) | 25(7.4%) |  |
| No | 462(90.8%) | 313(92.6%) |  |
| Blindness or Severe Visual Impairment |  |  | 0.703 |
| Yes | 137(26.9%) | 95(28.1%) |  |
| No | 372(73.1%) | 243(71.9%) |  |
| Deafness or Severe Hearing Impairment |  |  | 0.417 |
| Yes | 167(32.8%) | 120(35.5%) |  |
| No | 342(67.2%) | 218(64.5%) |  |
| Muteness or Severe Speech Impairment |  |  | 0.558 |
| Yes | 4(0.8%) | 4(1.2%) |  |
| No | 505(99.2%) | 334(98.8%) |  |
| Hypertension |  |  | <0.001 |
| Yes | 239(47.0%) | 95(28.1%) |  |
| No | 270(53.0%) | 243(71.9%) |  |
| Dyslipidemia |  |  | <0.001 |
| Yes | 89(17.5%) | 16(4.7%) |  |
| No | 420(82.5%) | 322(95.3%) |  |
| Diabetes or Elevated Blood Sugar |  |  | 0.162 |
| Yes | 45(8.8%) | 21(6.2%) |  |
| No | 464(91.2%) | 317(93.8%) |  |
| Cancer |  |  | 0.219 |
| Yes | 12(2.4%) | 4(1.2%) |  |
| No | 497(97.6%) | 334(98.8%) |  |
| Chronic Lung Disease |  |  | 0.209 |
| Yes | 112(22.0%) | 87(25.7%) |  |
| No | 397(78.0%) | 251(74.3%) |  |
| Liver Disease |  |  | 0.492 |
| Yes | 46(9.0%) | 26(7.7%) |  |
| No | 463(91.0%) | 312(92.3%) |  |
| Heart Disease |  |  | <0.001 |
| Yes | 133(26.1%) | 55(16.3%) |  |
| No | 376(73.9%) | 283(83.7%) |  |
| Stroke |  |  | 0.938 |
| Yes | 25(4.9%) | 17(5.0%) |  |
| No | 484(95.1%) | 321(95.0%) |  |
| Kidney Disease |  |  | 0.766 |
| Yes | 76(14.9%) | 53(15.7%) |  |
| No | 433(85.1%) | 285(84.3%) |  |
| Stomach Diseases |  |  | 0.299 |
| Yes | 218(42.8%) | 157(46.4%) |  |
| No | 291(57.2%) | 181(53.6%) |  |
| Emotional or Mental Problems |  |  | 0.914 |
| Yes | 10(2.0%) | 7(2.1%) |  |
| No | 499(98.0%) | 331(97.9%) |  |
| Memory Disorders |  |  | 0.793 |
| Yes | 23(4.5%) | 14(4.1%) |  |
| No | 486(95.5%) | 324(95.9%) |  |
| Asthma |  |  | 0.235 |
| Yes | 46(9.0%) | 39(11.5%) |  |
| No | 463(91.0%) | 299(88.5%) |  |
| Cataract Surgery |  |  | 0.656 |
| Yes | 26(5.1%) | 15(4.4%) |  |
| No | 483(94.9%) | 323(95.6%) |  |
| Use of Hearing Aids |  |  | 0.415 |
| Yes | 1(0.2%) | 0(0%) |  |
| No | 508(99.8%) | 338(100%) |  |
| Glaucoma |  |  | 0.993 |
| Yes | 12(2.4%) | 8(2.4%) |  |
| No | 497(97.6%) | 330(97.6%) |  |
| Complete Tooth Loss |  |  | <0.01 |
| Yes | 103(20.2%) | 99(29.3%) |  |
| No | 406(79.8%) | 239(70.7%) |  |
| Smoking History |  |  | 0.610 |
| Yes | 178(35.0%) | 124(36.7%) |  |
| No | 331(65.0%) | 214(63.3%) |  |
| Headaches |  |  | 0.253 |
| Yes | 284(55.8%) | 202(59.8%) |  |
| No | 225(44.2%) | 136(40.2%) |  |
| Shoulder Pain |  |  | 0.965 |
| Yes | 314(61.7%) | 208(61.5%) |  |
| No | 195(38.3%) | 130(38.5%) |  |
| Arm Pain |  |  | 0.071 |
| Yes | 263(51.7%) | 196(58.0%) |  |
| No | 246(48.3%) | 142(42.0%) |  |
| Wrist Pain |  |  | 0.096 |
| Yes | 204(40.1%) | 155(45.9%) |  |
| No | 305(59.9%) | 183(54.1%) |  |
| Finger Pain |  |  | 0.339 |
| Yes | 209(41.1%) | 150(44.4%) |  |
| No | 300(58.9%) | 188(55.6%) |  |
| Chest Pain |  |  | <0.01 |
| Yes | 137(26.9%) | 123(36.4%) |  |
| No | 372(73.1%) | 215(63.6%) |  |
| Stomach Pain |  |  | 0.082 |
| Yes | 188(36.9%) | 145(42.9%) |  |
| No | 321(63.1%) | 193(57.1%) |  |
| Back Pain |  |  | 0.041 |
| Yes | 236(46.4%) | 181(53.6%) |  |
| No | 273(53.6%) | 157(46.4%) |  |
| Waist Pain |  |  | 0.522 |
| Yes | 374(73.5%) | 255(75.4%) |  |
| No | 135(26.5%) | 83(24.6%) |  |
| Hip Pain |  |  | 0.731 |
| Yes | 148(29.1%) | 102(30.2%) |  |
| No | 361(70.9%) | 236(69.8%) |  |
| Leg Pain |  |  | 0.673 |
| Yes | 353(69.4%) | 239(70.7%) |  |
| No | 156(30.6%) | 99(29.3%) |  |
| Ankle Pain |  |  | 0.090 |
| Yes | 202(39.7%) | 154(45.6%) |  |
| No | 307(60.3%) | 184(54.4%) |  |
| Toe Pain |  |  | 0.113 |
| Yes | 137(26.9%) | 108(32.0%) |  |
| No | 372(73.1%) | 230(68.0%) |  |
| Neck Pain |  |  | 0.501 |
| Yes | 211(41.5%) | 148(43.8%) |  |
| No | 298(58.5%) | 190(56.2%) |  |
| Nap Duration (h) | 0.62±0.75 | 0.48±0.70 | <0.01 |
| Participation in Social Activities |  |  | 0.121 |
| Yes | 246(48.3%) | 145(42.9%) |  |
| No | 263(51.7%) | 193(57.1%) |  |
| History of Alcohol Consumption |  |  | 0.615 |
| Yes | 214(42.0%) | 148(43.8%) |  |
| No | 295(58.0%) | 190(56.2%) |  |
| Difficulty in Jogging |  |  | 0.827 |
| Yes | 432(84.9%) | 285(84.3%) |  |
| No | 77(15.1%) | 53(15.7%) |  |
| Difficulty Walking 1 km |  |  | 0.167 |
| Yes | 246(48.3%) | 147(43.5%) |  |
| No | 263(51.7%) | 191(56.5%) |  |
| Difficulty Walking 100m |  |  | 0.167 |
| Yes | 246(48.3%) | 147(43.5%) |  |
| No | 263(51.7%) | 191(56.5%) |  |
| Difficulty in Standing Up |  |  | 0.094 |
| Yes | 349(68.6%) | 213(63.0%) |  |
| No | 160(31.4%) | 125(37.0%) |  |
| Difficulty Climbing Stairs |  |  | 0.160 |
| Yes | 402(79.0%) | 253(74.9%) |  |
| No | 107(21.0%) | 85(25.1%) |  |
| Difficulty Bending |  |  | 0.180 |
| Yes | 384(75.4%) | 241(71.3%) |  |
| No | 125(24.6%) | 97(28.7%) |  |
| Difficulty Reaching Upwards |  |  | 0.118 |
| Yes | 156(30.6%) | 121(35.8%) |  |
| No | 353(69.4%) | 217(64.2%) |  |
| Difficulty Lifting 10 kg |  |  | <0.01 |
| Yes | 172(33.8%) | 150(44.4%) |  |
| No | 337(66.2%) | 188(55.6%) |  |
| Difficulty Picking Up Coins from a Table |  |  | 0.880 |
| Yes | 60(11.8%) | 41(12.1%) |  |
| No | 449(88.2%) | 297(87.9%) |  |
| ADL |  |  | 0.902 |
| Yes | 213(41.8%) | 140(41.4%) |  |
| No | 296(58.2%) | 198(58.6%) |  |
| Primary Cooking Fuel |  |  | <0.01 |
| Coal | 50(9.82%) | 19(5.62%) |  |
| Natural Gas | 45(8.84%) | 22(6.51%) |  |
| Marsh Gas | 5(0.98%) | 7(2.07%) |  |
| Liquefied Petroleum Gas | 47(9.23%) | 15(4.44%) |  |
| Electric | 72(14.15%) | 53(15.68%) |  |
| Crop Residue/Wood Burning | 285(55.99%) | 220(65.09%) |  |
| Other | 5(0.98%) | 2(0.59%) |  |
| Depression |  |  | <0.01 |
| Yes | 374(73.5%) | 275(81.4%) |  |
| No | 135(26.5%) | 63(18.6%) |  |
| Cognition | 10.35±5.61 | 8.72±5.55 | <0.001 |
| Self-Rated Health |  |  | 0.956 |
| Very Good | 2(0.39%) | 2(0.59%) |  |
| Good | 14(2.75%) | 8(2.37%) |  |
| Fair | 139(27.31%) | 93(27.51%) |  |
| Poor | 277(54.42%) | 185(54.74%) |  |
| Very poor | 77(15.13%) | 50(14.79%) |  |
