# Supplementary material for: An explainable machine learning-based prediction model for sarcopenia in elderly Chinese people with knee osteoarthritis
Source: Aging Clin Exp Res. 2025 Mar 7;37(1):67. doi: 10.1007/s40520-025-02931-x (PMC11889032; doi:10.1007/s40520-025-02931-x)
Supplement: Supplementary file 2 — Supplementary Material 2 [file 40520_2025_2931_MOESM2_ESM.docx]

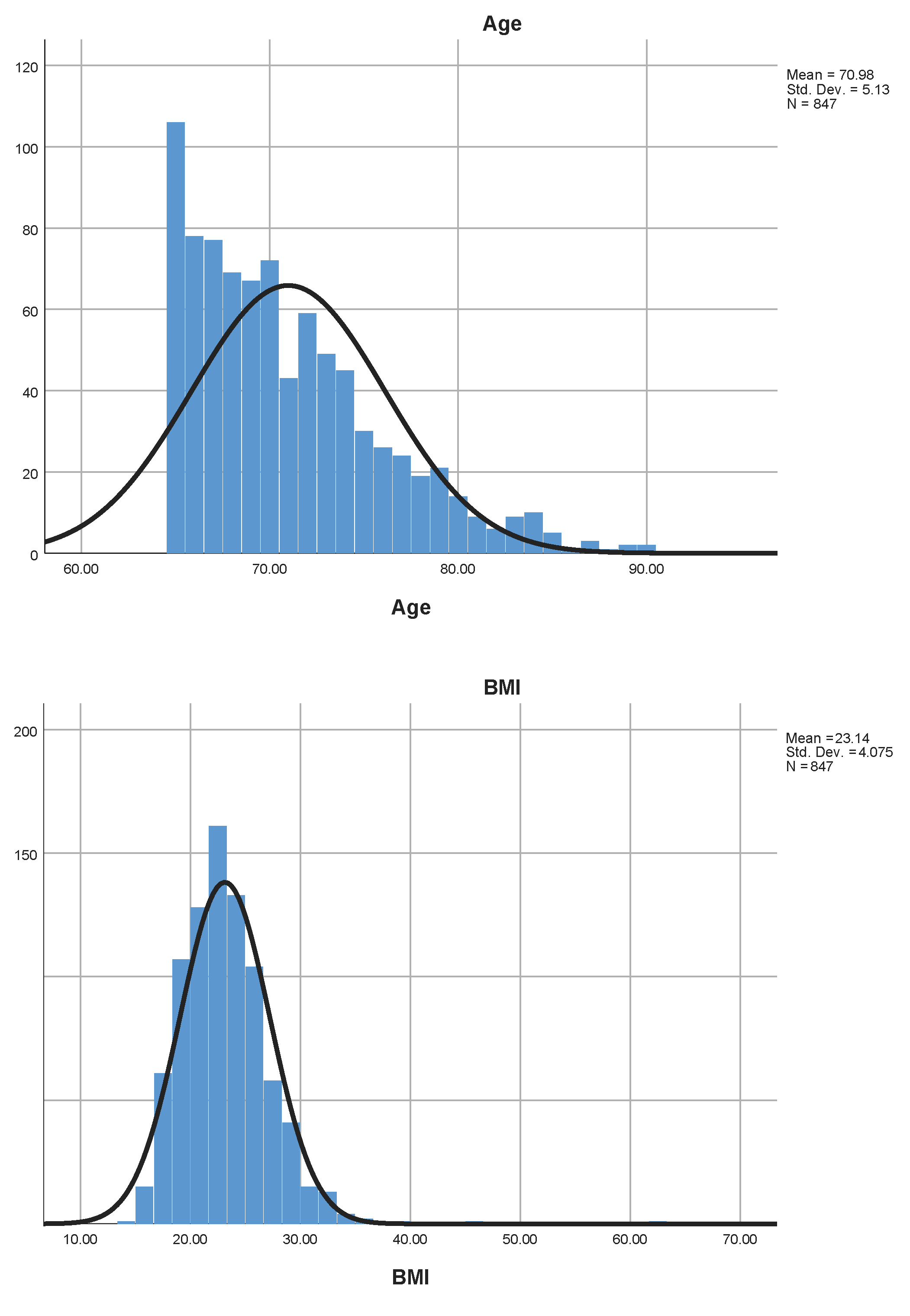

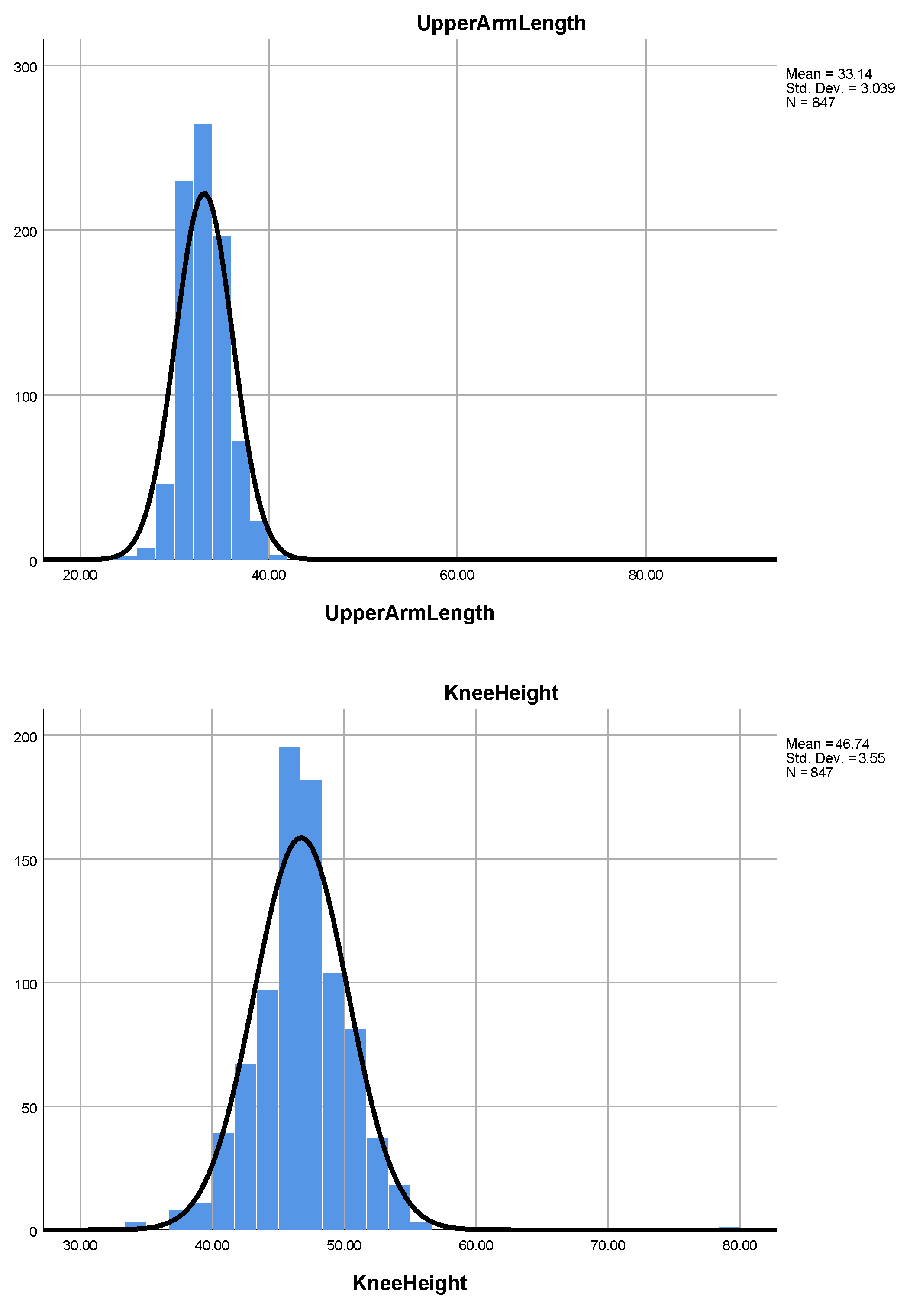


Fig.1. Histograms of the following variables: Age, BMI, Upper Arm Length, Knee Height.


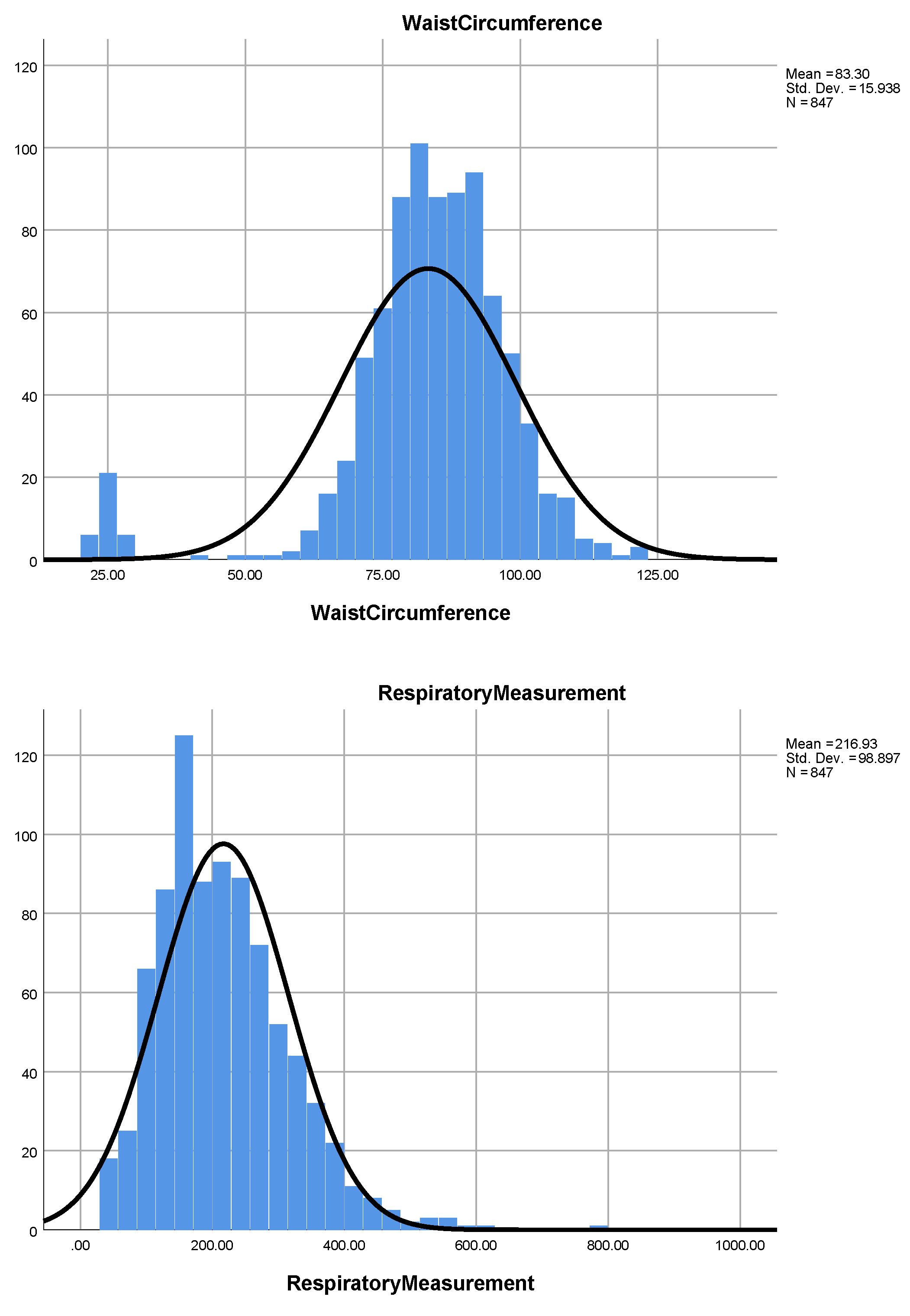

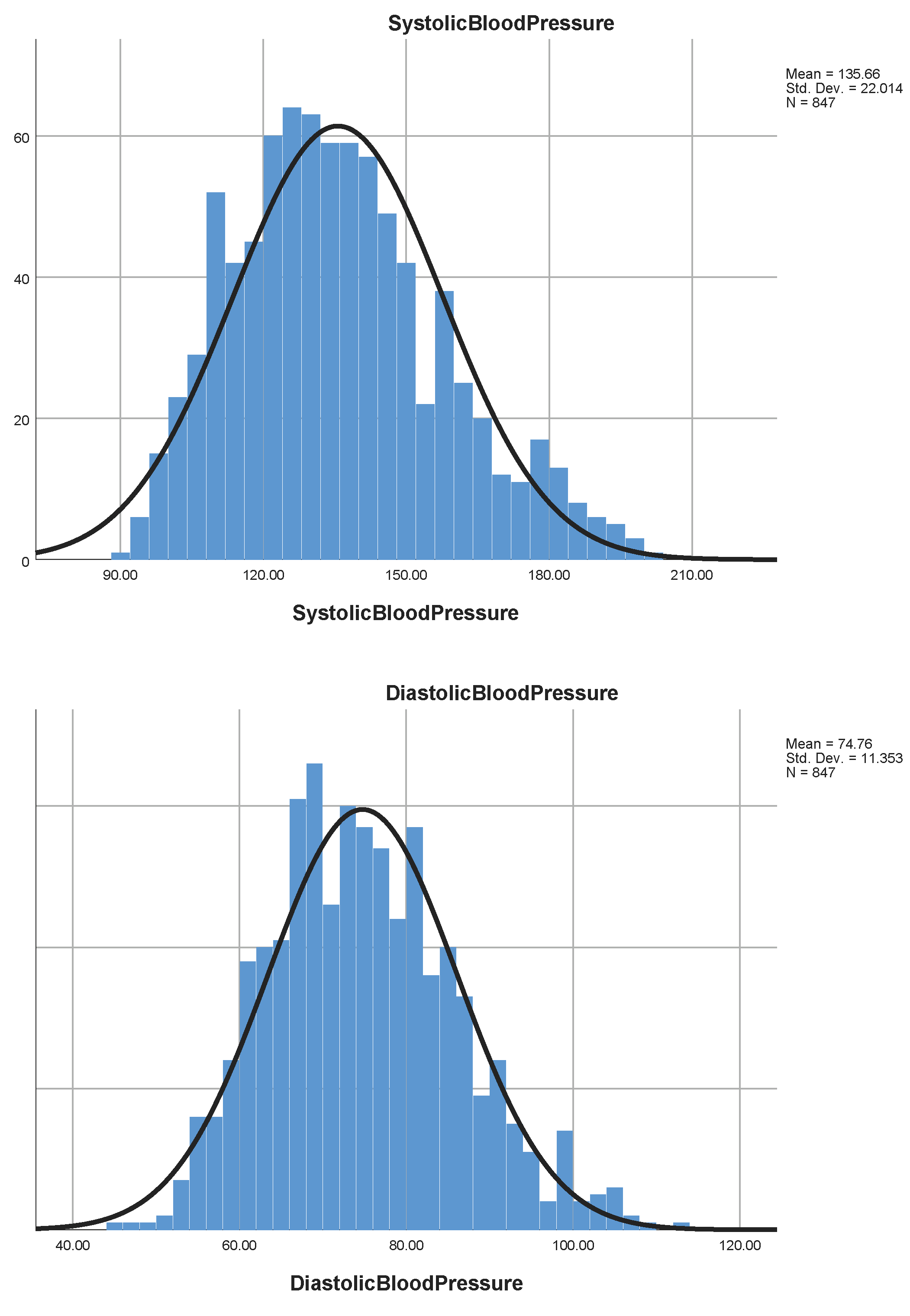


Fig.2. Histograms of the following variables: Waist Circumference, Respiratory Measurement, Systolic Blood Pressure, Diastolic Blood Pressure.


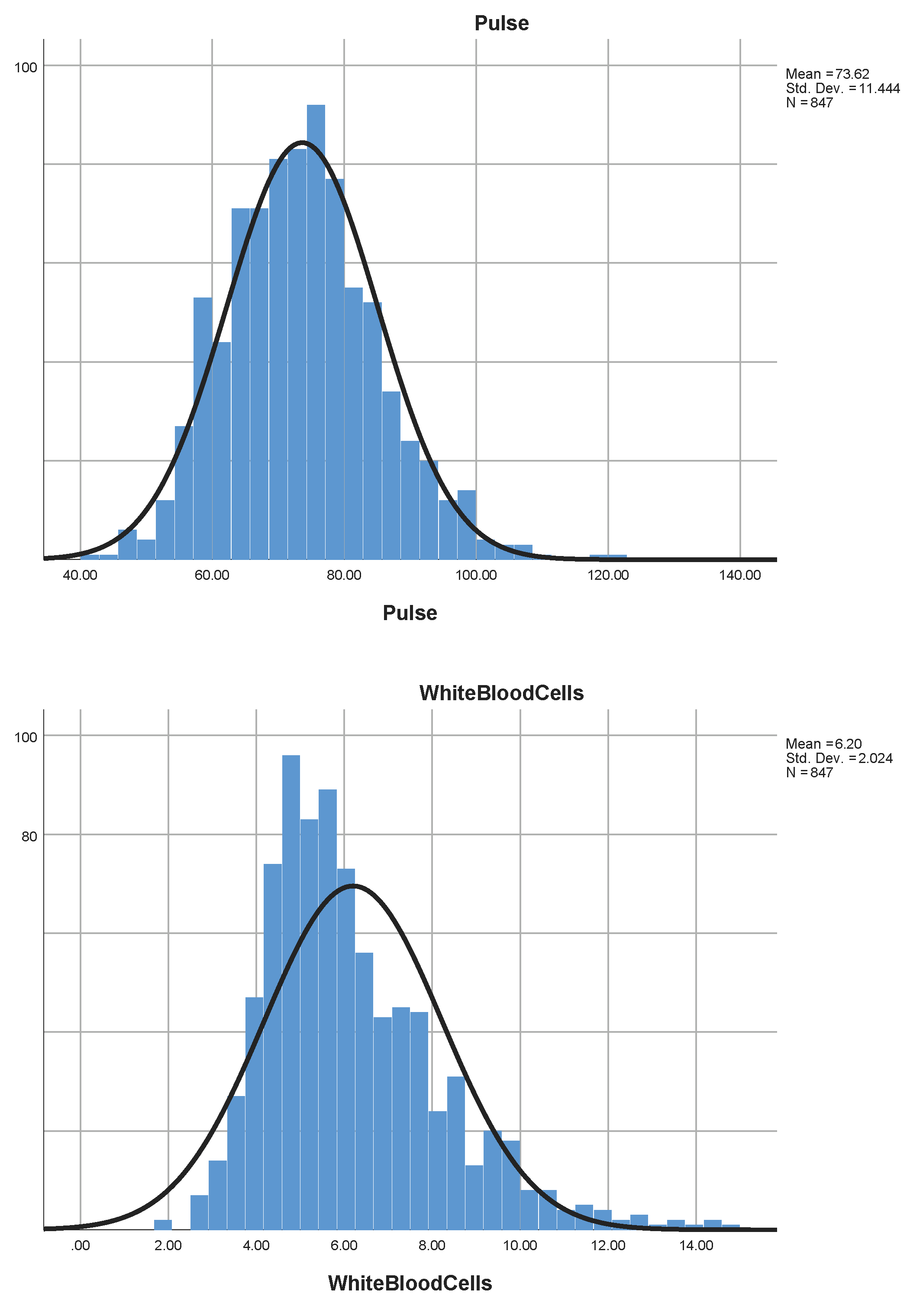

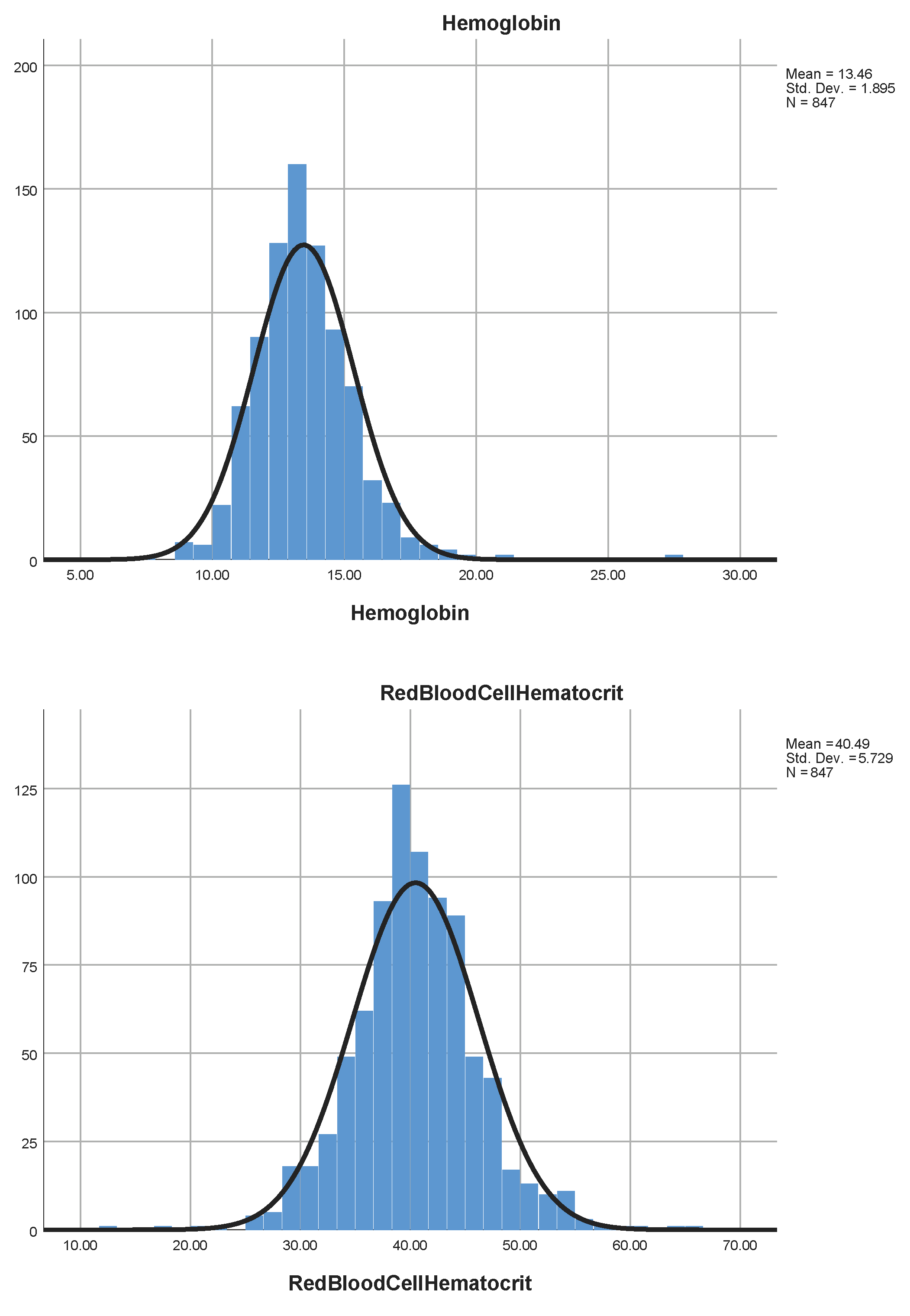


Fig.3. Histograms of the following variables: Pulse, White Blood Cells, Hemoglobin, Red Blood Cell Hematocrit.


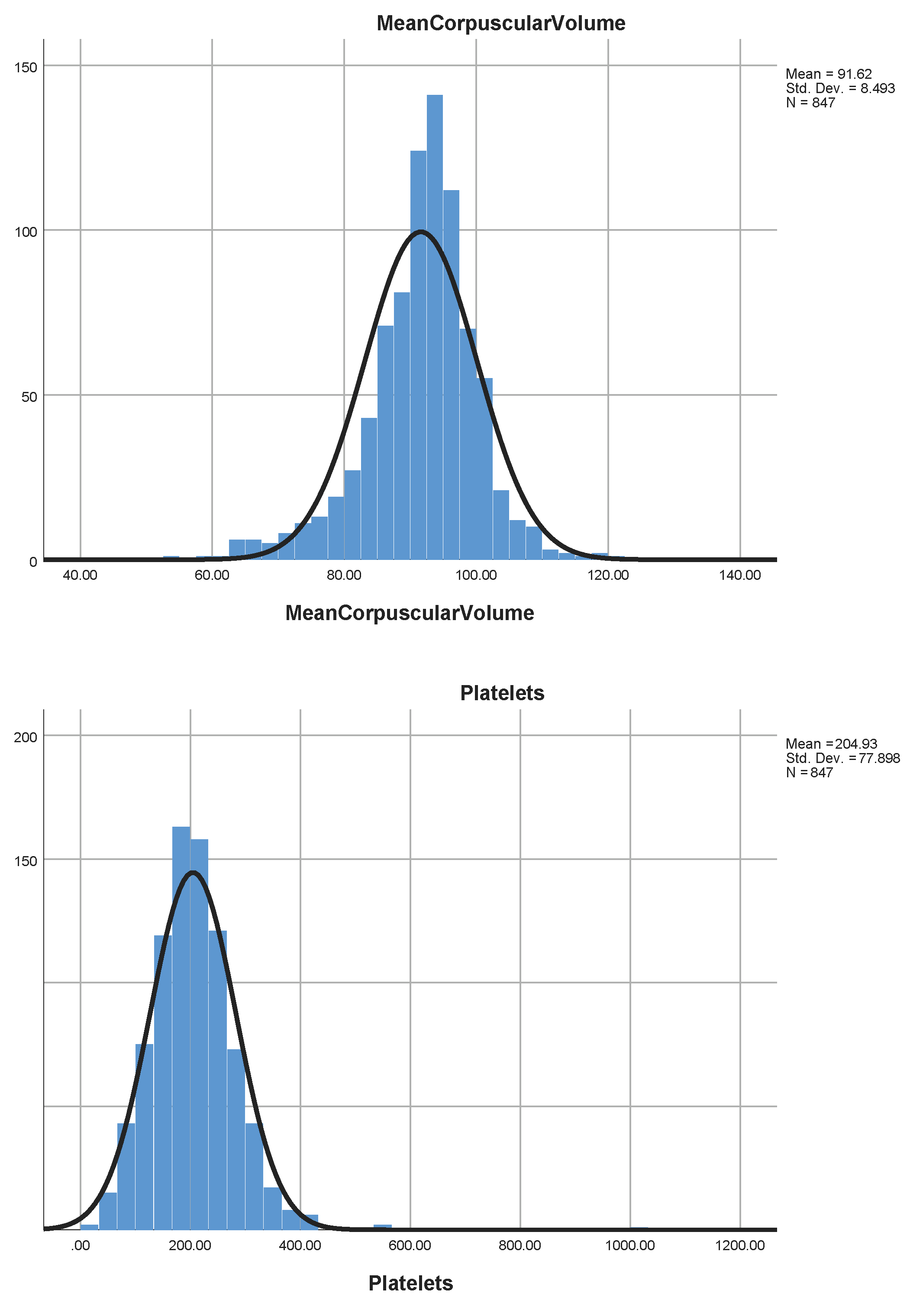

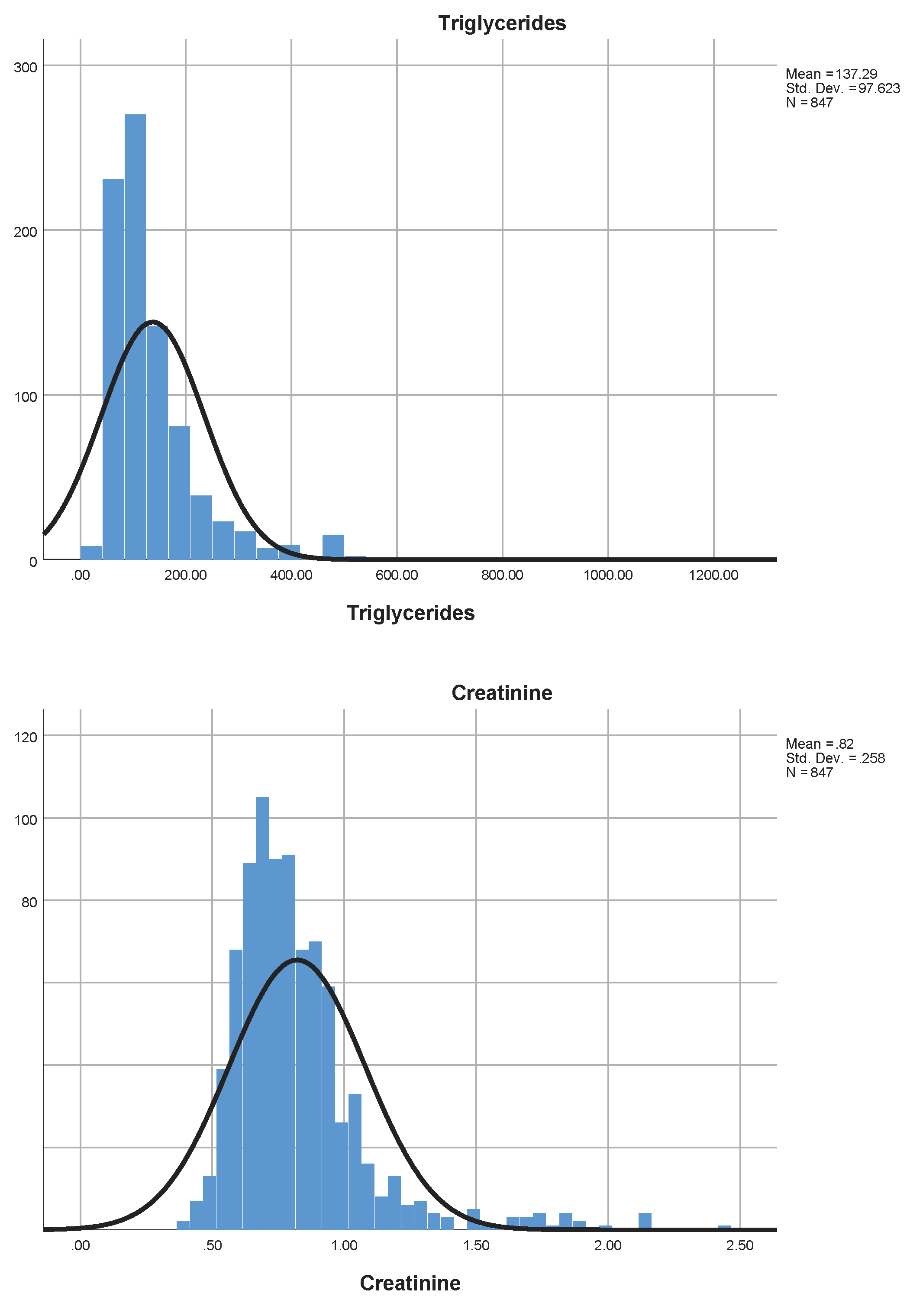


Fig.4. Histograms of the following variables: Mean Corpuscular Volume, Platelets, Triglycerides, Creatinine.


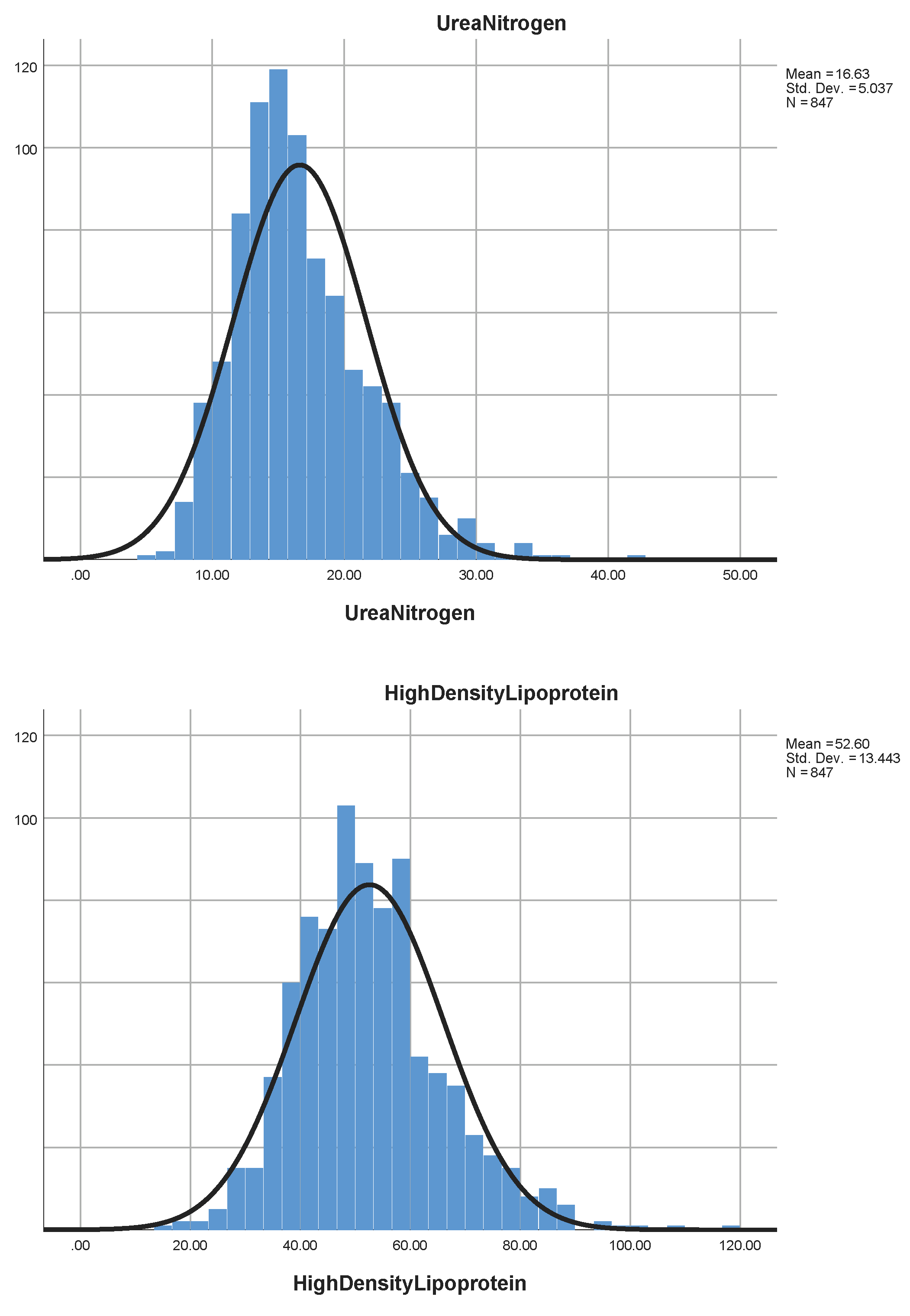

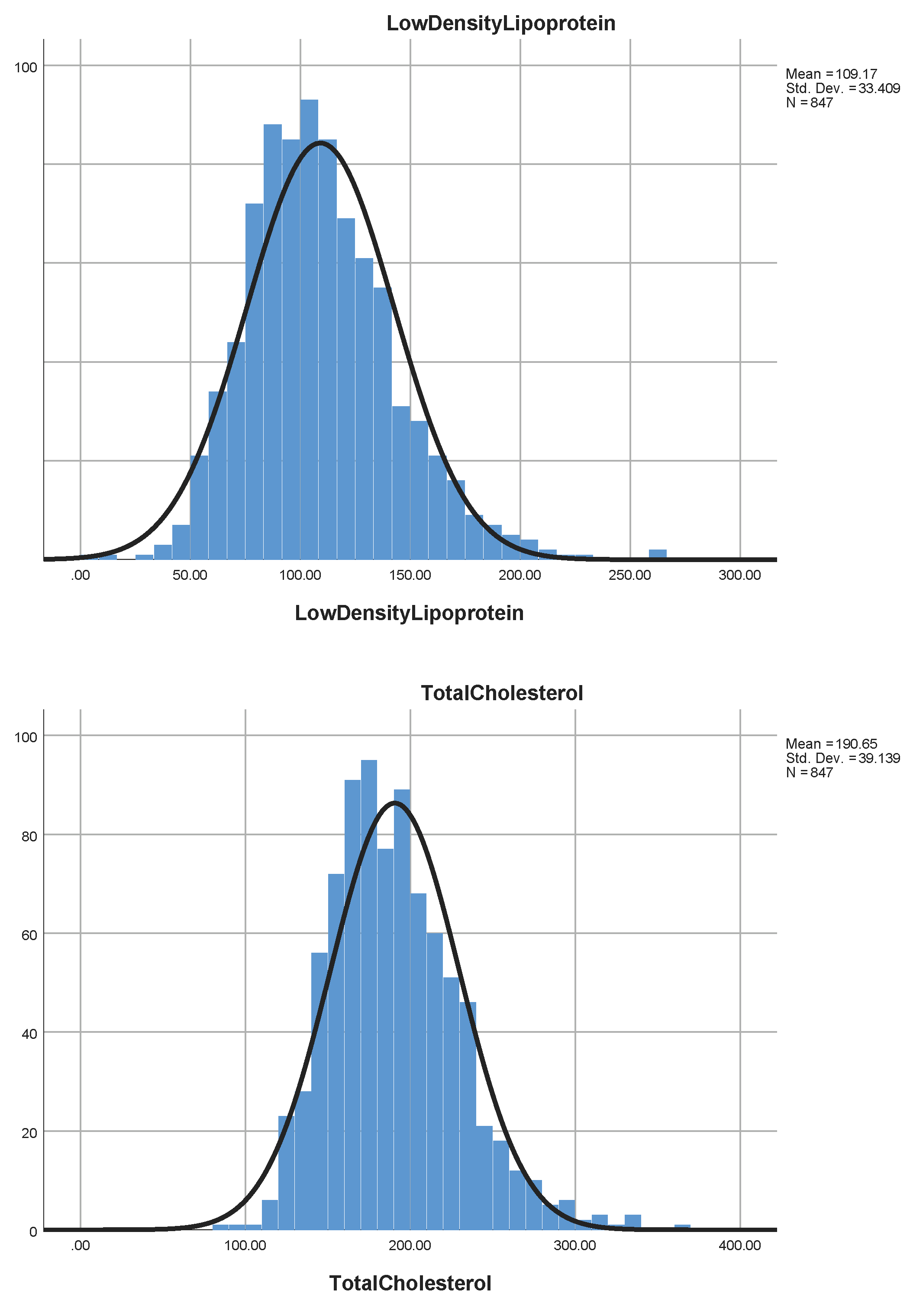


Fig.5. Histograms of the following variables: Urea Nitrogen, High-Density Lipoprotein, Low-Density Lipoprotein, Total Cholesterol.


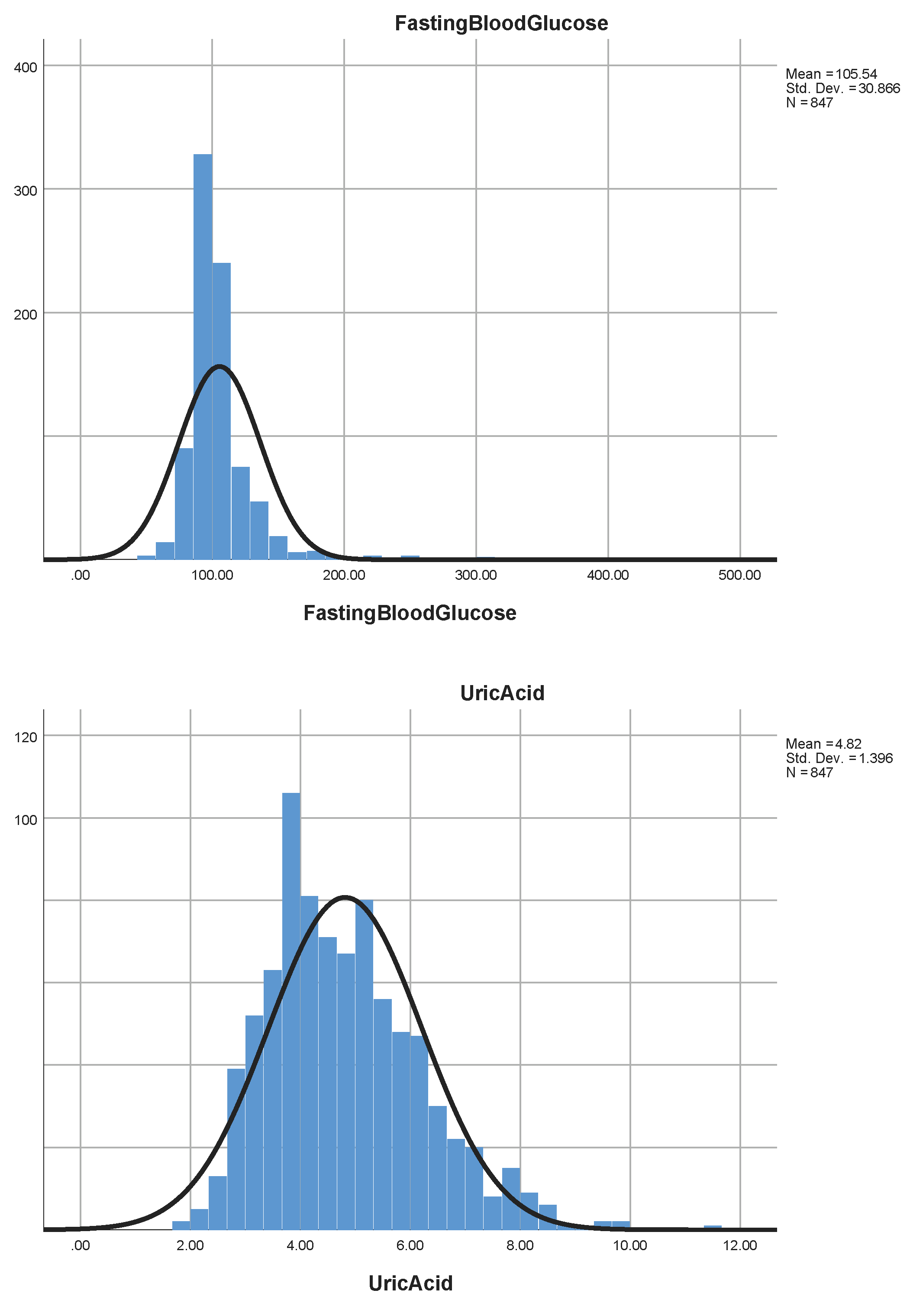

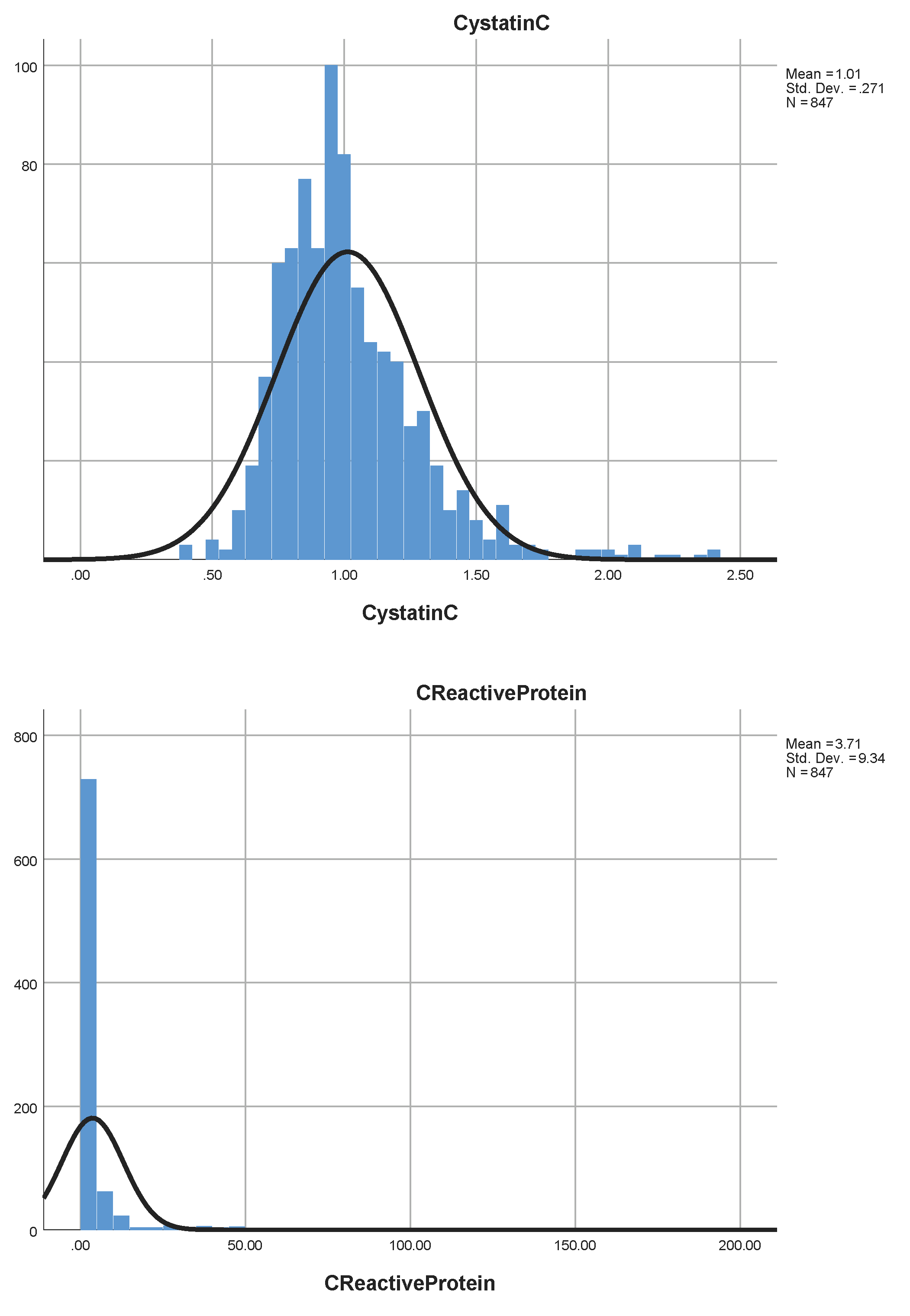


Fig.6. Histograms of the following variables: Fasting Blood Glucose, Uric Acid, Cystatin C, C-Reactive Protein.


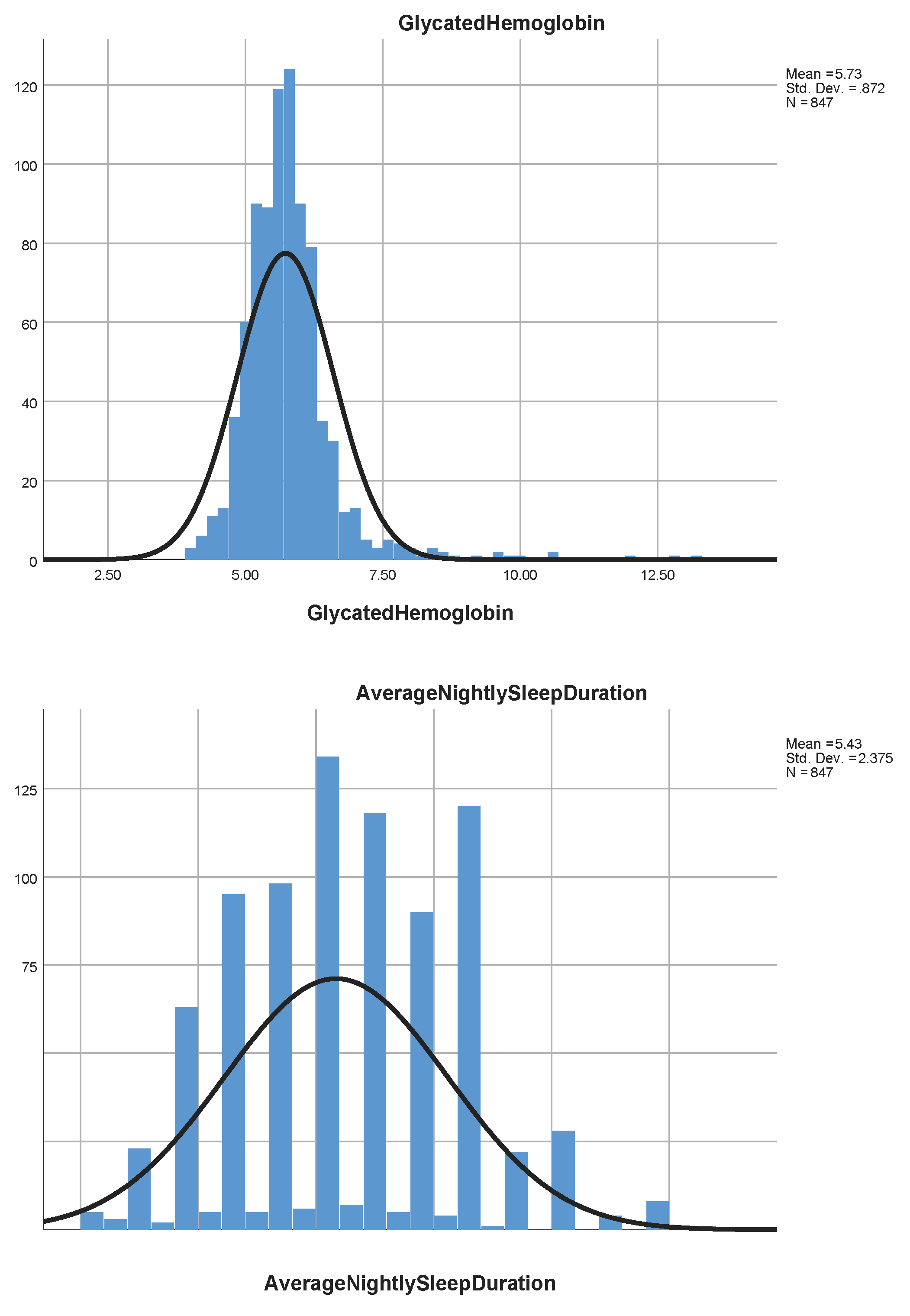

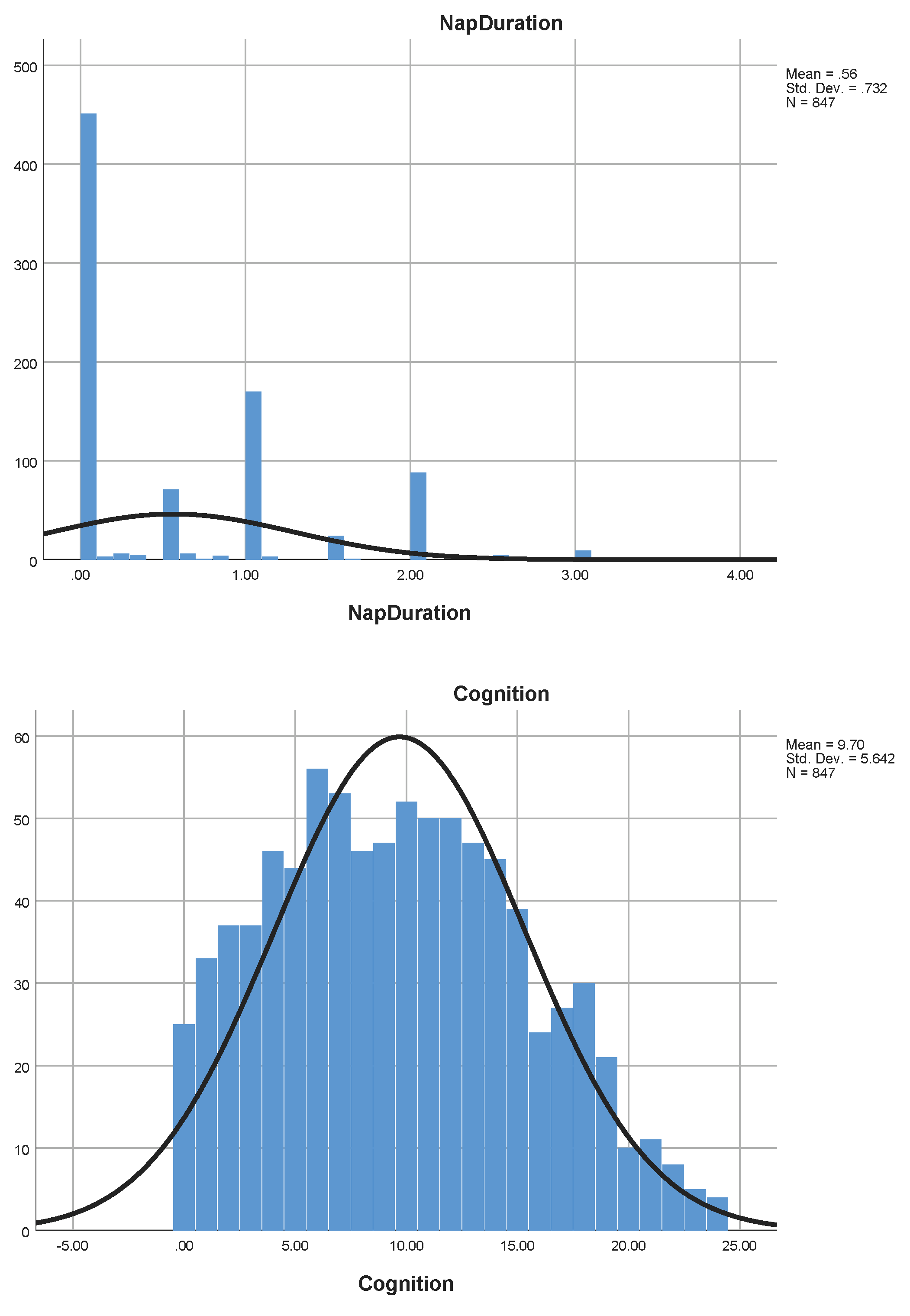
\

Fig.7. Histograms of the following variables: Glycated Hemoglobin, Average Nightly, Sleep Duration, Nap Duration, Cognition.


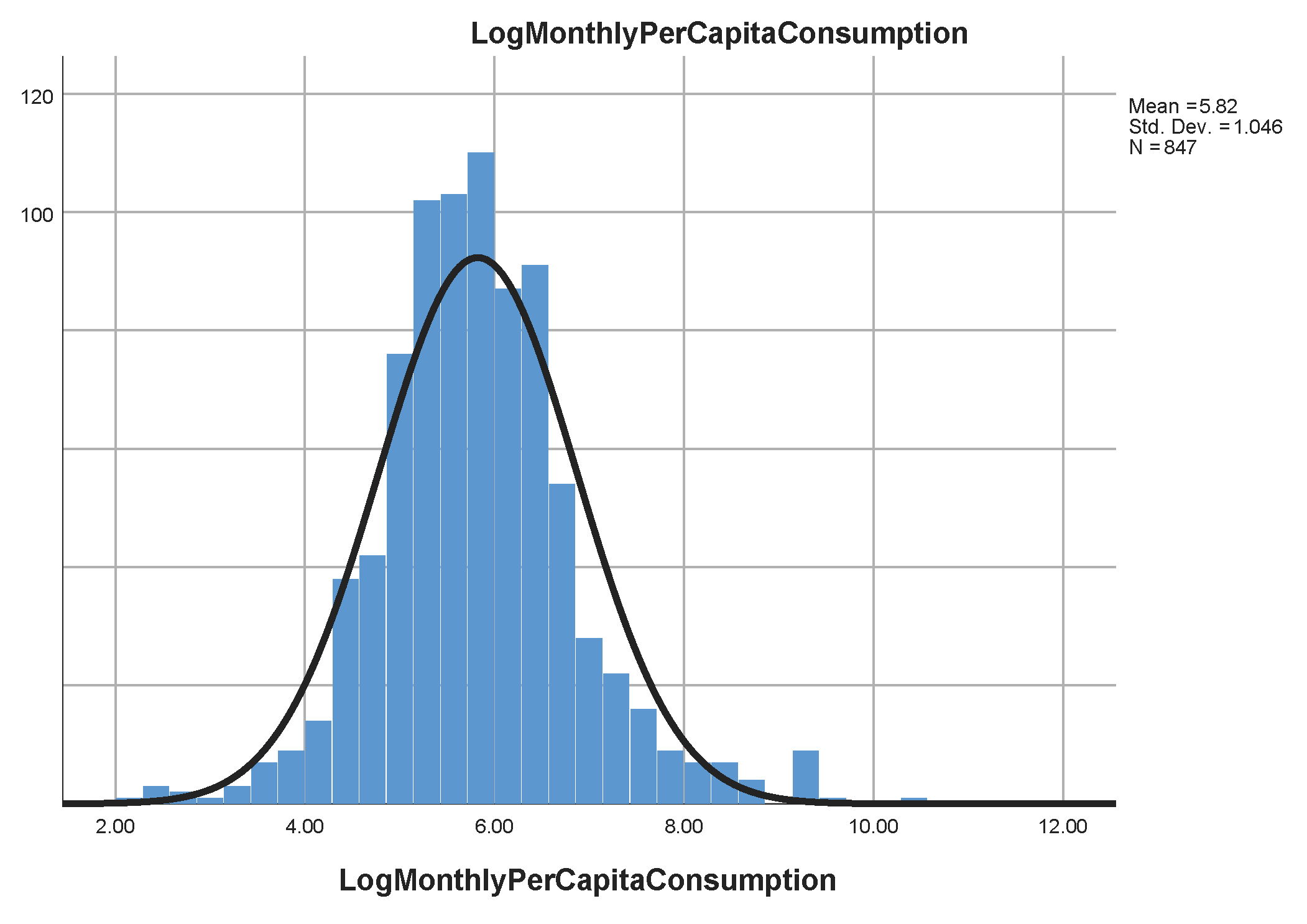


Fig.8. Histograms of the following variables: Log Monthly Per Capita Consumption.
